# Tapered Optical Fibre Sensors: Current Trends and Future Perspectives

**DOI:** 10.3390/s19102294

**Published:** 2019-05-17

**Authors:** Sergiy Korposh, Stephen W. James, Seung-Woo Lee, Ralph P. Tatam

**Affiliations:** 1 Department of Electrical and Electronic Engineering, The University of Nottingham, University Park, Nottingham NG7 2RD, UK; 2 Centre for Engineering Photonics, Cranfield University, Cranfield, Bedfordshire MK43 0AL, UK; s.w.james@cranfield.ac.uk (S.W.J.); r.p.tatam@cranfield.ac.uk (R.P.T.); 3 Department of Chemical Process and Environment, Graduate School of Environmental Engineering, The University of Kitakyushu, Kitakyushu 808-0135, Japan; leesw@kitakyu-u.ac.jp

**Keywords:** tapered optical fibre sensors, evanescent wave spectroscopy, modal interferometry, whispering gallery mode, functional nano-thin coatings

## Abstract

The development of reliable, affordable and efficient sensors is a key step in providing tools for efficient monitoring of critical environmental parameters. This review focuses on the use of tapered optical fibres as an environmental sensing platform. Tapered fibres allow access to the evanescent wave of the propagating mode, which can be exploited to facilitate chemical sensing by spectroscopic evaluation of the medium surrounding the optical fibre, by measurement of the refractive index of the medium, or by coupling to other waveguides formed of chemically sensitive materials. In addition, the reduced diameter of the tapered section of the optical fibre can offer benefits when measuring physical parameters such as strain and temperature. A review of the basic sensing platforms implemented using tapered optical fibres and their application for development of fibre-optic physical, chemical and bio-sensors is presented.

## 1. Introduction

Accurate understanding of the environment is important for proper functioning of various processes associated with human activities, ranging from the dependence of wellbeing on the quality of the indoor and outdoor environments [1,2] to complex industrial processes [3]. This requires the monitoring and control of critical environmental parameters, which can broadly be divided into physical (temperature, strain, pressure, etc.) and bio-chemical (concentration of contaminants and bio-hazardants). A recent publication [3] provided a comprehensive review on the use of optical fibre sensors for environmental monitoring in civil, petroleum and agricultural engineering. This paper focuses on the use of tapered optical fibre sensors for the measurement of parameters important in environmental monitoring.

Optical sensors detect changes in optical parameters (refractive index (RI), absorbance, reflectance, fluorescence, etc.) that depend upon the physicochemical parameters (pressure, strain, temperature, chemical composition, etc.) of the investigated environment. Optical fibres offer a convenient method for creating optical sensors, directing light to, and collecting light from, the measurement region, so called extrinsic sensors [4] or using the fibre itself as the transducer, so called intrinsic sensors [5]. Fibre optic sensors possess several advantages over conventional sensor techniques [5,6,7]. They are not susceptible to electromagnetic interference, they can survive harsh environments and tolerate high temperatures. They are biocompatible and are readily multiplexed, allowing the simultaneous monitoring of a number of measurands. They can be used for remote monitoring of the environment because of the low attenuation that is a property of light propagation in telecommunications grade single mode optical fibres.

Optical fibre-based measurement techniques have attracted a great deal of attention in a variety of analytical areas such as chemical and biological sensing, environmental and structural health monitoring and medical diagnosis. The large variety of designs and measurement schemes that may be implemented using optical fibres provides the potential for the creation of highly sensitive and selective sensors for deployment in real world environments.

One of the simplest methods for the fabrication of optical fibre sensor elements is based on the tapering of a relatively short section (of length ranging from sub-millimetre to tens of millimetres) of the optical fibre. This provides access to the evanescent wave (EW) of the mode propagating through the tapered region, facilitating interaction with the surrounding medium and allowing the measurement of parameters such as RI or chemical composition. A tapered optical fibre consists of a region of fibre with reduced and uniform diameter (the waist) that is bounded by conical sections where the diameter of the fibre changes to merge the tapered section with the unperturbed surrounding fibre. The optical properties of the tapered fibre waveguide are influenced by the profile of the conical tapering sections, by the diameter of the taper waist and by the RI of the surrounding medium. The proportion of the power in the EW, and thus the interaction with the surrounding medium, increases with decreasing diameter of the taper waist and with decreasing difference between the RI of the fibre and surrounding medium [8,9]. Tapered optical fibres offer a number of attractive features for sensor development, including large evanescent fields, flexibility and compactness. In the case of a tapered optical fibre that is coated with a functional material, the properties of the waveguide are influenced by the optical thickness (product of RI, and geometrical thickness) of the coating.

Originally, tapered optical fibres were employed for the development of directional couplers, where two or more tapers are fused together, as they provide efficient light coupling between fibres [10]. More recently, tapered optical fibres have also found applications in sensor development [11], polarizers, submicron wire [12], light amplifiers [13] and near and far field microscopy [14].

### Fabrication of Tapered Optical Fibres

**Figure 1:**
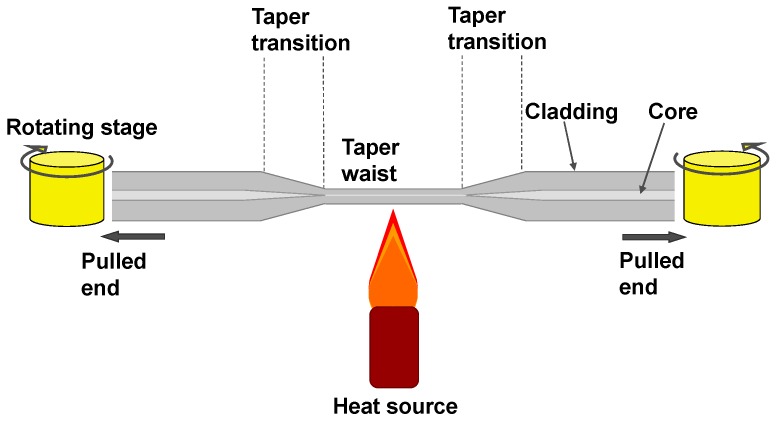
Schematic illustration of the flame approach used for the fabrication of tapered optical fibres.

The fabrication of tapered optical fibres is generally achieved by heating a short section of the fibre while simultaneously pulling the two ends of the fibre, as illustrated in Figure 1. The heat source could be the flame of a gas burner [8], high power laser radiation [15,16], or an arc discharge [17]. In single mode optical fibre, the one of the functions of the cladding is to reduce the penetration of the electric field of the propagating mode into surrounding medium. In the tapering process, the core and cladding diameters are reduced by the same proportion. This process leads to coupling of light from the fundamental mode of the untapered fibre to modes of the tapered section that can interact with the surrounding medium. The other way to facilitate the interaction of the light propagating within the optical fibre with the surrounding medium is to thin the cladding of the optical fibre, leaving the dimensions of the core unchanged. This can be achieved by chemical etching, polishing and focused ion beam etching [18]. The thinning of the cladding increases the interaction of the EW of the propagating mode with the surrounding medium. In contrast to tapered fibres, there is no mode coupling between fundamental and higher order modes. The key difference between two configurations is explained in Section 2.3 and presents the mechanism for coupling of the fundamental core mode to higher order cladding modes in tapered fibres, while no such phenomenon is observed in thinned cladding optical fibres. This difference results in the different sensitivities, with tapered optical fibre more sensitive enabling more information to be extracted about the surrounding medium, as explained later in this review. In this review, we will focus on tapered optical fibres, i.e., optical fibres in which the core and cladding diameters are reduced by the same proportion, rather than on optical fibres with thinned claddings.

Figure 1 shows the “heat and stretch” approach to tapered optical fibre fabrication. The polymer buffer coating (typically acrylate) is removed from a section in the middle of a length of optical fibre using a mechanical stripper or suitable solvent. The optical fibre tails on either side of the section to be tapered are then fixed on translation stages. The buffer coating-removed section is exposed to the heat source while the fibre tails are pulled in opposite directions. Using this approach, optical fibre tapers of different diameters with well-defined taper waists can be fabricated. The optical characteristics of a tapered optical fibre are determined by the taper diameter and by the geometry of the taper transition region. If the transition regions satisfies the adiabaticity criteria, which requires that the taper transition length is longer than the beat length between the fundamental and 2nd order modes and thus a lower angle taper [19], there is no energy coupling to higher order modes. Such adiabatic tapers are characterised by low losses and single mode operation. In non-adiabatic tapers, where this criterion is not satisfied, the coupling of light to higher order modes increases the losses of the taper, and also introduces features into the transmission spectrum that are caused by interference between the propagating modes, which can be used for sensing applications.

The evanescent field distribution surrounding the tapered region can be measured directly using a scanning near-field optical microscope, which can produce results that are in good agreement with finite difference beam propagation analysis [20].

**Figure 2:**
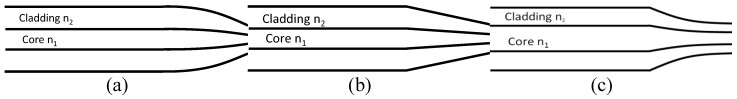
Geometry of a (**a**) parabolic (**b**) linear and (**c**) exponential–linear taper profiles [23]. Reprinted with permission. Copyright 2003 Elsevier.

As stated, the geometry of the tapered fibre plays a crucial role in its performance as a sensor, with smaller diameter taper waists providing the higher sensitivity [21,22,23,24]. Typical taper profiles are shown in Figure 2. 

It has been shown that, for a given heating profile, the shape of a tapered fibre is independent of the material properties and the stretching conditions applied at the fibre ends [25]. Thus, control of a taper’s shape can be achieved simply by using different heating profiles, achieved for example by scanning the heat source along the section of the fibre to be tapered. 

Birks and Li [24] introduced the procedure for analysing the hotzone length variation required to produce a given shape of taper. They have demonstrated that an optimal adiabatic taper can be made using a traveling burner tapering system. 

Control of the heating of the fibre within the heating zone is of critical importance for producing tapers with uniform waists. In this regard, a CO_2_ laser offers a number of advantages over the use of a flame such as the heated-zone properties can be controlled in a repeatable fashion, the production of air turbulence and combustion contaminants can be avoided [16,26].

**Figure 3:**
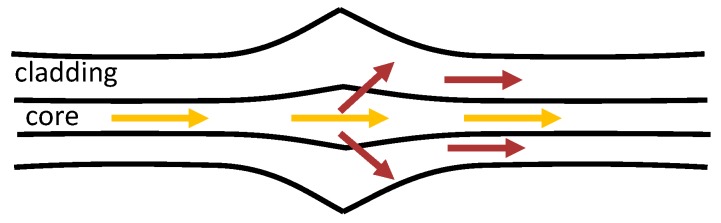
A schematic diagram of a waist enlarged tapered optical fibre.

In addition to tapered fibres with reduced diameter, that creates two cones (biconical tapers), there has been interest in waist enlarged tapers (WET) where the diameter of core and cladding are increased at the overlap during the fusion splicing of two optical fibres (Figure 3). One of the advantages of WET over down-stretched tapers is the increased mechanical stability of the device [21]. 

Bending of the tapered optical fibres into a U-shape or an S-shape allows to increase the interaction of the evanescent wave with the surrounding medium thus increasing sensitivity [27,28,29,30]. 

## 2. Tapered Optical Fibre Sensors

Tapered optical fibre sensors were used in different applications such as environmental monitoring, healthcare and structural health monitoring [31,32,33,34,35,36]. Typically, two methods can be employed to measure the change associated with the interaction between the EW and the medium of interest [37]. The first approach exploits detection of the light losses within the tapered region, which can be caused by both the change in spectral properties of the analyte and RI of the surrounding medium [38]. The second method utilises interferometric method in which the change of the effective refractive indices of the modes propagating through the taper, is measured by using mode coupling devices such as in-fibre gratings, surface plasmon resonances [39] and lossy mode resonances [40].

### 2.1. Evanescent Wave Spectroscopy

Chemical composition of the environment surrounding the optical fibre can be measured quantitatively and qualitatively using evanescent wave spectroscopy which is a highly sensitive and powerful technique [37]. The penetration depth (*d_p_*) of the EW decays exponentially with the distance from the interface between the waveguide and the surrounding environment and is described by [4]:

(1) $$d_{p}=\frac{\lambda }{2\pi {\left(n_{eff}^{2}-n_{s}{}^{2}\right)}^{1/2}},$$

where *λ* is the wavelength of light in free space, *n_s_* is the RI of the surrounding environment and *n_eff_* is the effective RI of the mode guided by the optical fibre. 

The chemical composition of the surrounding medium influences the propagation of the EW, and thus of the propagating mode, according to the Lambert–Beer law

(2) $$log\frac{I}{I_{0}}=c\times \alpha \times L,$$

where *α* is the molar extinction coefficient, *c* is the concentration of the chemical compound, *L* is the optical path-length, and *I* and *I*_0_ are the light intensities before and after interaction with chemical compound, respectively. 

The spectroscopy and hence chemical composition of the surrounding medium can be analysed simply by detecting the transmitted light by coupling the output from the distal end of the optical fibre into a spectrometer. It is important to match the parameters of the light source and spectrometer such as emission wavelength and sensitivity range with the absorption properties of the particular compound that needs to be detected. Typically, the absorption features of environmental pollutants are located in the infrared and mid-infrared spectral range (2.5–10 μm) which limits the application of silica optical fibres since they are not transparent in this spectral window. The low attenuation of chalcogenide glasses in the infrared region, where specific absorption features of organic molecules are located, makes the use of the low losses of tapered chalcogenide optical fibre attractive for evanescent wave spectroscopy [41].

**Figure 4:**
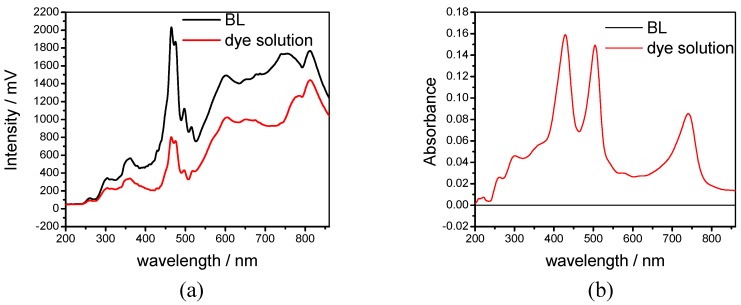
(**a**) Transmission spectrum of a hard-clad multimode silica optical fibre with the plastic cladding removed (black line), and after (red line) immersion into a porphyrin dye compound; (**b**) absorption spectrum calculated from Figure 4a [37,42]; Reprinted from [37] under creative common free licence (http://creativecommons.org/licenses/by/4.0/).

Figure 4 shows a typical example of the evanescent wave spectroscopy that measured absorption features of the conducted a porphyrin dye compound using a hard-clad multimode silica optical fibre (core diameter, 200 ± 5 µm; cladding diameter 225 ± 5 µm, coating diameter 500 ± 30 µm, Pure Silica/TECS Hard Cladding Tefzel, FT200UMT, Thorlab) with a section of the plastic cladding removed [37]. Figure 4b demonstrates the absorption features of the porphyrin dye compounds located at ca. 420, 494 and 710 nm. 

### 2.2. Radiation Losses and Scattering

For some of the modes in an optical fibre, the guidance conditions are not satisfied owing to the reduction of the size of the cladding and the core diameters caused by the tapering of the optical fibre, resulting in radiation losses, i.e., leakage of these modes from the fibre into the surrounding medium. The magnitude of the radiation losses depends strongly on the RI of the surrounding medium. This mechanism has been employed for the development of an RI sensor with a resolution of 7 × 10^−4^ RIU [43].

### 2.3. Modal Interferometry

Modal interferometry (MI) is a sensitive and powerful approach in sensor development that exploits the interaction between lower and higher order modes in the fibre region. Those modes propagate with different effective refractive indices and respond to the analyte differently resulting in the change in phase that is measured on the mode recombination. 

Approaches based on, for example, in-fibre gratings, non-adiabatic tapers, regions of core mismatch and directional couplers may be employed to create regions where two modes are excited and subsequently recombined to interfere [44,45]. Interference between modes results in the channelled spectrum measured by spectrometer and the phase of the channelled spectrum dependents on the difference in the optical path lengths of the interfering modes, according to [37,46]:

(3) $$\varphi =\frac{2\pi }{\lambda }\left(\delta n_{eff}\right)L,$$

where *λ* is the free-space wavelength, *L* is the centre-to-centre distance between two coupling elements and *δn*_eff_ is the difference in effective refractive indices between higher order and fundamental modes. 

**Figure 5:**
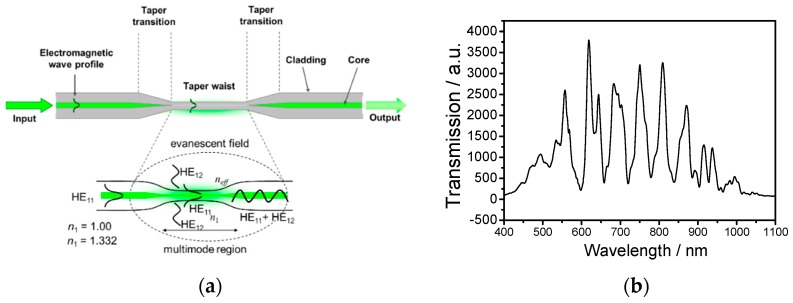
(**a**) Schematic illustration of the structure of a tapered optical fibre; HE_11_ fundamental core mode coupled to higher order modes HE_12_ at the first taper transition region; higher order modes are then combined with the fundamental core mode at second transition region HE_11_ + HE_12;_ and (**b**) a typical channelled transmission spectrum of the non-adiabatic taper with waist diameter of 10 μm fabricated in an optical fibre with a cut-off wavelength of 670 nm (Fibrecore SM670). Reprinted from [37] under creative common free licence (http://creativecommons.org/licenses/by/4.0/).

Depending on the taper geometry, optical fibre tapers can be divided into adiabatic and non-adiabatic types: if the angle of the taper transition region is small and the cylindrical symmetry of the optical fibre is retained, resulting in most of the optical power remaining in the fundamental mode the tapers are considered adiabatic; if the optical power is coupled into higher order modes the tapers are non-adiabatic tapers. For non-adiabatic tapers of diameter less than 10 μm, the linearly polarised, LP_01_, mode of the single mode fibre generally couples to the HE_11_ and HE_12_ modes of the tapered waist [35,47], which propagate with different effective indices along the taper waist and interfere when recombine at the second taper transition (Figure 5a). The corresponding channelled spectrum is shown in Figure 5b. 

In general, two interrogation approaches can be employed for tapered optical fibre sensors: (i) measurement of the dependence of the amplitude of the signal on the particular measurand [8] and (ii) dependence of the wavelengths of the features in the channelled spectrum on the particular measurand [48]. It should be noted that first approach requires a reference measurement to compensate for any drift in the light source intensity and for any losses induced by the bending of the optical fibre. 

### 2.4. Gratings in Tapered Optical Fibres

**Figure 6:**
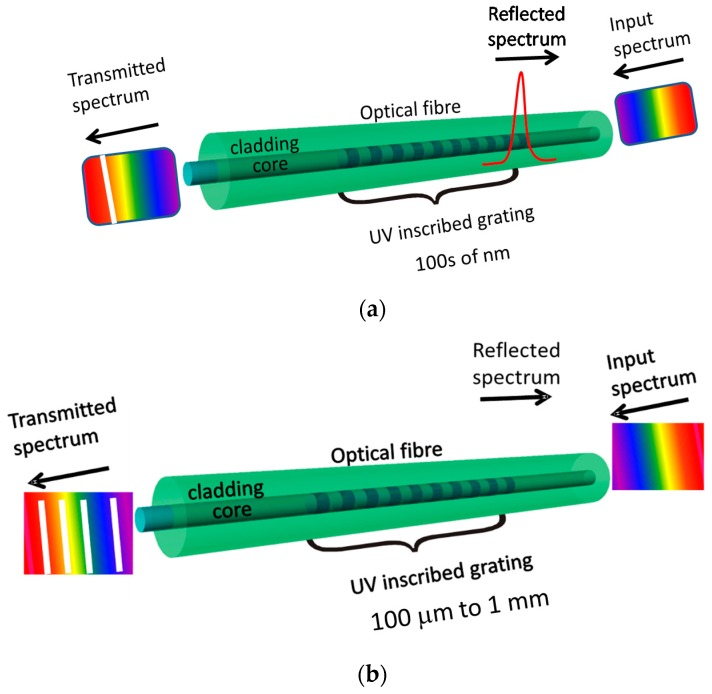
Schematic illustrations of: (**a**) a fibre Bragg grating (FBG) and (**b**) a long period grating (LPG). Reprinted from [37] under creative common free licence (http://creativecommons.org/licenses/by/4.0/).

Introduction of the periodic modulation of the refractive index inside the core of the optical fibre by exposure to a spatially modulated intensity pattern from a UV or femtosecond laser creates a diffraction grating, which can be used for sensor development. Generally, optical fibre grating sensors are classified as fibre Bragg grating (FBG) (reflection type gratings; Figure 6a) and a long period grating (LPG) (transmission type gratings; Figure 6b) with the grating periods of 100 s of nm and 100 μm to 1 mm, respectively [37,49].

Optical fibre grating sensors are sensitive to parameters that can influence the period of the grating or RI of the fibre. Fibre Bragg grating sensors are typically sensitive to strain and temperature [50,51]. Since in FBG all optical power remains in the core they are generally not sensitive to RI. To sensitise FBGs to the surrounding RI, an access to the EW needs to be obtained and this can be achieved by polishing, etching or tapering optical fibre. Changes in the surrounding RI induces the Bragg wavelength shifts and modulates the reflected power [37].

In an LPG the position of the resonance bands in the transmission spectrum is dependent upon product of the period of the LPG and the difference between the core and cladding mode indices, which makes them inherently sensitive to the surrounding RI, and to the optical thickness of nanoscale coatings deposited onto the cladding [37,49]. 

**Figure 7:**
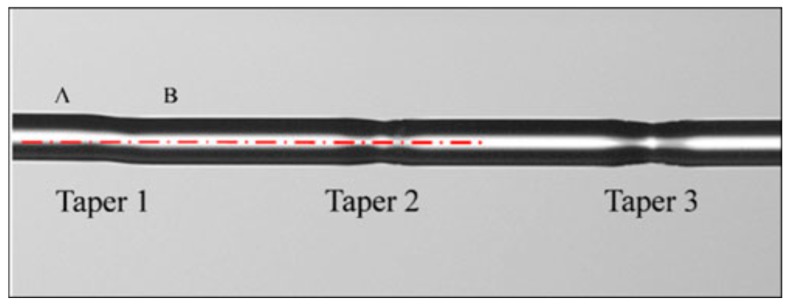
Micrograph of a tapered long period grating TLPG with three micro-tapers. Taper 1 has a distorted profile. The fibre at position A is offset from the fibre axis compared to position B. The centre axis of the fibre is shown as a dashed red line. SM750 fibre was used (magnification × 10); Reprinted with permission from [55]. Copyright 2018 Elsevier.

While LPGs are predominantly sensitive to strain temperature, curvature and surrounding RI [52], the combination of tapers with LPGs can offer an increase in sensitivity and functionality via enhancement of the interaction of the EW with the surrounding medium. Periodic tapering of an optical fibre using CO_2_ laser as a heating source can be used as an alternative to UV laser based inscription methods for LPG fabrication, as shown in Figure 7 [53,54] with ultra-short (<400 micron length) tapered LPGs recently demonstrated using CO_2_ laser fabrication [55].

A further approach to LPG fabrication, in which the tapered section of an optical fibre is embedded into a polymeric periodic structure using soft mold-replica lithography has been demonstrated [56] or side-contacted with metal grating [57]. It was observed that the best performance (−1.328 nm/°C and 21 nm/RIU) obtained for polymeric structures is when a 10 mm long grating with the period of 400 µm, and a waist diameter of 20 µm is used. An advantage of the polymeric grating over metallic is the flexibility of fabrication, which allows for the optical fibre to be embedded in the grating and eliminates needs of further adjustments [56].

The wavelength-encoded nature of the grating based sensors offers a number of advantages; not least, the ability to multiplex a serial array of FBG or LPG sensors in a single optical fibre by ensuring that each has a different period, and thus a different resonance wavelength.

### 2.5. Surface Plasmon Resonance 

**Figure 8:**
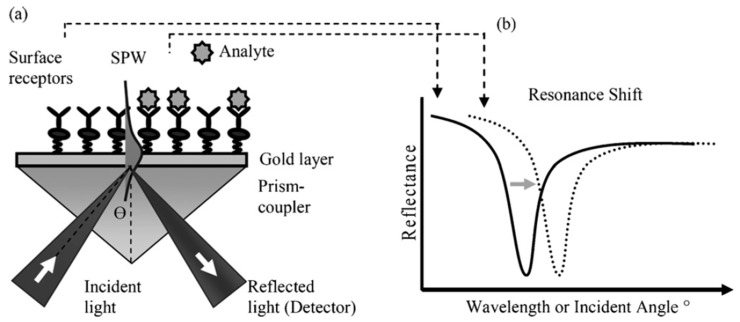
(**a**) Schematic illustration of the surface plasmon resonance (SPR) sensor configuration employing prism for surface plasmon excitation; and (**b**) shift in resonance features (wavelength or incidence angle) caused by the binding event [59], (Reprinted with permission. Copyright 2007 Elsevier).

Surface plasmon resonance (SPR) is a powerful tool, used extensively for chemical, biological and medical applications, that belongs to the group of refractometric sensing devices that measure changes in the RI in the field of an EW [58] (Figure 8). 

The analyte-induced change in optical parameters at the interface between the gold coating and the surrounding medium modulates the resonant coupling of incident light to the propagating surface plasmon wave [59]. The existence of the surface plasmon wave is dictated by the electromagnetic properties of the metal (typically gold or silver)–dielectric (sample medium) interface [59]. The characteristics of the resonant coupling provide information about compounds bound to the metal layer. This information is tracked by monitoring either the wavelength at which coupling occurs at a fixed angle of incidence, the angle of incidence at which coupling occurs at a particular wavelength or the intensity of the light at a fixed angle and wavelength. 

In the typical SPR configuration, the so-called Kretchmann arrangement, a prism coated with a metal layer is employed, creating a sensor that requires bulk optics and expensive mechanical parts. Optical tapered fibre based SPR, in which a tapered fibre optic is coated with a thin metal film, offers advantages over the standard SPR configuration owing to its small size, light weight and automatic alignment, with no compromise in terms of sensitivity and overall sensor performance [39].

## 3. Applications of Tapered Optical Fibre Sensors

### 3.1. Refractometry—Single Taper Devices

Refractive index is a fundamental property of any material, which depends on environment parameters such as temperature, pressure, humidity and concentration of any chemicals present. The measurement of RI can provide valuable information about these important environmental parameters. It should be noted, that measurements of the RI does not allow to discriminate what chemicals are present in the medium. 

As was mentioned above, generally, two parameters of the transmission spectrum can be measured to reveal information on changes of the external RI; the wavelength shift of the interference features in the transmission spectrum of a non-adiabatic taper, or the attenuation (radiation losses) of the light transmitted through the tapered waist. Typically, the performance of optical fibre refractometers is tested in the 1.333–1.360 range, corresponding to the typical refractive indices of biological fluids. Villatoro reported a sensor based on surrounding-refractive-index-induced radiation losses in a tapered multimode optical fibre with a waist diameter of 60 μm, which was capable of measuring RI in the range 1.36–1.46, with a limit of detection below 10^−4^ RIU [60].

Modal interferometers based on non-adiabatic tapered optical fibre (NATOF) sensors can be used to measure changes in the RI of a solution, and thus the concentration of an analyte, without specificity, by monitoring changes in the phase of the channelled spectrum. For instance, a taper of diameter 7 μm was used to measure the concentration of D-glucose in deionized water. The limit of detection of the NATOF was 55 ppm for D-glucose concentrations ranging from 0 to 80 mg/mL, and the limit of detection of the RI measurement corresponding to these concentrations, which lay in the range from 1.3330 to 1.3447, was 8.2 × 10^−^^6^ RIU [61]. 

NATOF sensors can also be used to monitor RI changes induced by bio-molecular interactions in biological systems. The response of an NATOF was used to show that the interactions of various groups of amino acids (AA), such as L-alanine, L-leucine and L-cysteine with D-glucose, sucrose and water molecules, depend on functional groups such as OH, H, CH_2_*,* NH^3+^ and COO^−^. Such studies can improve the understanding of the interactions between AA molecules and entities present in biological matrices, without the requirement to monitor their spectroscopy [61].

The RI change caused by the presence of the *Escherichia coli* (JM101 strain) in solution at concentrations of 100, 1000, 7000 and 7 million cells/mL in water were measured using a 5.5 μm diameter taper [22,34]. The measurement principle was based on the detection of the RI change induced by the presence of the bacteria at relatively high concentrations [22,34]. The study showed that the sensitivity increased with reducing taper diameter, which was attributed to the associated increase of the penetration of evanescent field into the surrounding medium [22]. 

Tapered optical fibres can be used for the quasi-distributed sensing of RI, using optical frequency domain reflectometry (OFDR) to interrogate an array of tapered sections created in series in a single optical fibre. Changes in the attenuation of the Rayleigh backscattered signal from the tapered regions were used to monitor the RI of the surrounding medium. An analysis of the performance of the system showed that, for tapers of diameter 50 μm, up to nine tapers could be multiplexed using commercial OFDR instrumentation [62].

The sensitivity of tapered optical fibre refractometers can be improved by deposition of a mesoporous coating of silica nanospheres onto the surface of the taper via covalent immobilization [63]. The sensitivity enhancement was attributed to the coupled effects of Mie scattering and multimode propagation. The presence of the scattering centres increases the power fraction of the evanescent wave and thus the interaction of the light with the surrounding medium, providing higher sensitivity as compared with non-scattering tapers [63]. The effect of the taper dimensions on sensor performance was studied and the sensitivity was improved by two orders of magnitude when a taper (thickness 2.8 μm and length 14 mm) was coated with a 400-nm thick film of silica nanospheres as compared with unmodified taper [63]. 

### 3.2. Grating Assisted Taper Devices

The combination of gratings with tapered fibres offers the prospect for the flexible design of devices with enhanced capabilities. The resonance bands in the transmission spectra of LPGs are inherently sensitive to the surrounding RI by virtue of the dependence of the resonance wavelength on the effective index of the cladding mode to which coupling occurs. The sensitivity of the LPG’s transmission spectrum to changes in surrounding RI can be increased by fabricating the LPG in the tapered region of an optical fibre [64]. The refractive index resolution achieved with this configuration was ±8.5 × 10^−5^ as compared to about 1 × 10^−4^ of the standard LPG optical fibre sensors [65].

**Figure 9:**
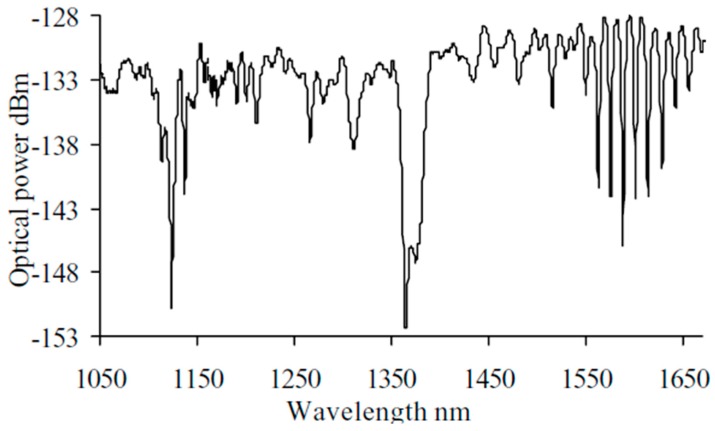
Transmission spectrum of LPG tapered fibre device; reprinted with permission from [66].

It is also possible to create modal interferometers by combining LPGs with tapers. For example, an LPG fabricated in a section of tapered optical fibre can be used to excite selectively cladding modes that subsequently interfere at the second transition region with the light that propagates through the core. This produces a channelled spectrum within the envelope of the resonance bands of the LPG, such as that shown in Figure 9. Rather than relying upon the fabrication of small diameter NATOFs, such modal interferometers can be created in tapers of larger diameter, which can be easier to fabricate and be more robust. An LPG with a grating period of 400 μm and of length 5 cm was fabricated in a biconical fibre taper with 34 μm diameter and 3.2 cm length made from standard step-index optical fibre, producing the channelled spectrum illustrated in Figure 9. A resolution of 1 × 10^−4^ RIU for refractive indices in the range of 1.30–1.34 was reported [66].

**Figure 10:**
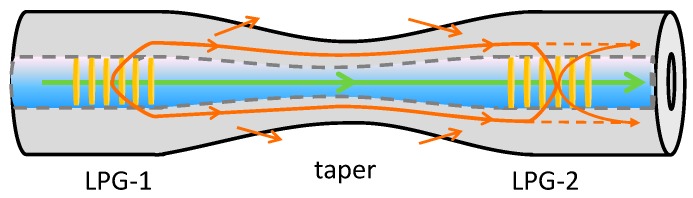
Schematic illustration of the fibre-taper cascaded LPG; arrows indicate coupling of the fundamental core mode (green arrow) to the cladding modes at LPG-1, their interaction with the surrounding medium at tapered region and recombination of higher order modes with the fundamental core mode at LPG-2 [67] (adapted from [67]).

Mach–Zehnder-like interference effects have been observed when LPGs with period larger than 300 μm were inscribed in a tapered fibre with a waist diameter of 25 μm. The devices exhibited a RI resolution of ±8.5 × 10^−^^5^ RIU for solutions with indices in the range of 1.330–1.335 [64]. The fabrication of a pair of identical, cascaded LPGs separated by a section of optical fibre to create an in-fibre Mach–Zehnder interferometer (MZI) has been investigated for sensing applications. The light coupled into the cladding by the 1st LPG is recoupled into the core by the 2nd LPG to interfere with the core mode, creating the interferometer. The sensitivity of such devices to changes in the surrounding RI can be increased if the section of fibre separating the LPGs is tapered, as illustrated in Figure 10, as the RI sensitivity of the cladding modes’ effective indices is enhanced [67]. A resolution of 5.8 × 10^−6^ RIU was achieved, assuming the measurement system has a spectral resolution of 1 pm. 

FBGs are generally insensitive to the RI of the medium surrounding the fibre cladding, unless fabricated in tapered optical fibres or in sections of fibre in which the cladding is thinned. FBG based refractometers can be interrogated by monitoring changes in the reflected wavelength or changes in the reflectivity. The intensity of the Bragg reflection from an FBG fabricated in a taper of diameter 30 μm was used to measure changes in the RI of the ambient environment as low as 2.5 × 10^−5^ RIU over a range of 1.450–1.456 RIU. A 150 pm shift of the Bragg wavelength was observed in response to an increase of the RI from 1.450 to 1.456, providing measurements with a resolution of 5 × 10^−6^ RIU when a spectral detection system with 1 pm resolution is used [68]. 

**Figure 11:**
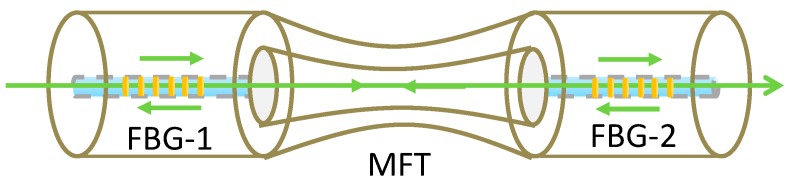
Schematic illustration of a refractive index (RI) sensor based on a multimode fibre taper splice between two lengths of single mode fibre, each of which contain an FBG (adapted from [69]).

One of the significant advantages offered by FBGs is the ability to wavelength-division-multiplex a serial array of FBGs in a single length of optical fibre. This was exploited to measure RI by splicing a tapered multimode fibre between two lengths of single mode fibre (SMF, Corning SMF28), each of which contained an FBG, as illustrated in Figure 11. Two FBGs (FBG1 and FBG2, Figure 11) with different Bragg wavelengths were used for signal demodulation. Changes in the RI influenced the attenuation of the tapered section of the fibre, which modified the intensity received from FBG2. The intensity difference between the two FBG signals was used to determine the change of RI of the medium surrounding the multimode fibre taper (MFT). Monitoring the Bragg wavelengths allowed the temperature to be measured. Experimental results showed that the sensor possessed a tailorable sensitivity to the change of the external RI by controlling the taper waist diameter [69].

### 3.3. Refractometry—Multi-Taper Devices

**Figure 12:**
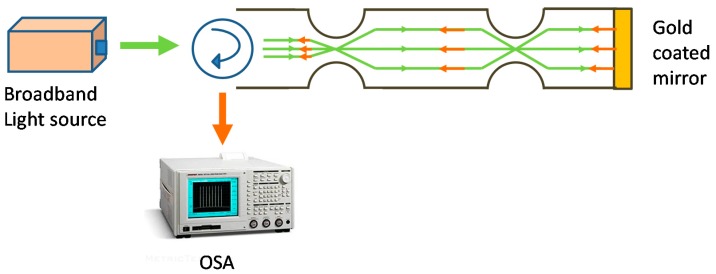
A schematic illustration of the inline double-pass Mach–Zehnder interferometer (MZI); arrows indicate excited cladding modes of the length of fibre separating the two tapers, creating different optical path lengths for higher order modes traveling in the cladding and fundamental core mode travelling in the core. Reprinted from [37] under creative common free licence (http://creativecommons.org/licenses/by/4.0/).

More complex tapered optical fibre configurations, employing multiple tapers, novel fibres and novel taper geometries, have also been investigated. For example, a double-pass in-line fibre taper MZI was formed in a single mode (Corning SMF28) optical fibre by creating two abrupt tapers using a fusion splicer (arc discharge). The tapers excited cladding modes of the length of fibre separating the two tapers, creating different optical path lengths for higher order modes traveling in the cladding and fundamental core mode travelling in the core (Figure 12) [70]. This is similar to the cascaded LPGs described earlier, but in this configuration, there is no control over the modes excited and the channelled spectrum is visible across the entire spectrum. The sensor can be interrogated by monitoring the light transmitted by the gold coating, where the light has traversed the sensing region once, or light reflected by the gold coating, which traverses the sensor twice. The resulting channelled spectrum was analysed to facilitate the measurement of surrounding RI and temperature. The temperature sensitivity arose from the thermo-optic effect and the thermal expansion of the MZI during the temperature change. The double-pass configuration increased the sensitivity compared to the single pass case because of the doubled interaction length between the propagating light and the measurands. The RI sensitivities of the single (measured in transmission without gold film) and double-pass MZIs were found to be 1.63 ± 0.01 × 10^5^ and 3.05 ± 0.01 × 10^5^ dBm/RIU, respectively, while the temperature sensitivities of single- and double-pass MZI were found to be 201.9 ± 2.7 and 382.7 ± 5.3 dBm/°C, respectively [70].

A similar configuration was used to implement an in-fibre Michelson interferometer (MchI) for RI measurements [71]. In the proposed MchI design, a single taper and a mirror at the tip of the optical fibre are employed. The modes excited in the cladding by the taper and the propagating core mode are reflected by the mirror and then recombine at the taper section. The experimental results show that a change in the RI of 10^−4^ caused a phase shift of ∼1.35° in the demodulated signal, given the use of an abrupt taper MchI with a sensing length of 20 cm.

**Figure 13:**
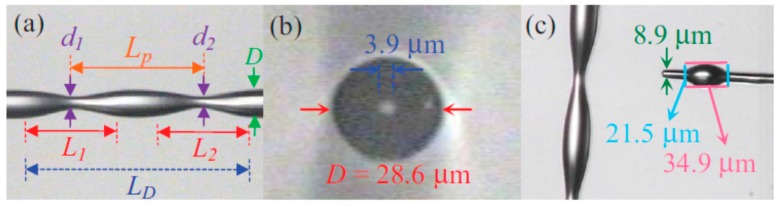
Photographs of (**a**) MZI with two micro-abrupt-tapers in a cladding-reduced Er/Yb codoped fibre; *L*_P_, length of the spanning fibre (between the centres of the adjacent abrupt tapers); and L_D_, lengths of the interferometer; L1, length of the first taper, L2 length of the second taper and d1 and d2 diameters of the taper one and two respectively; (**b**) cross-sectional views of the cladding-reduced Er/Yb co-doped fibre; and (**c**) 6.3-pL liquid drop is approaching the micro-abrupt-taper [72]. (© 2012 IEEE. Reprinted, with permission from [72]).

Microanalysis systems for early diagnosis and treatment of diseases, or for the detection of a single physiological cell or small specimens such as human embryonic stem cells, require a cellular-dimension sensing technique. Fibre sensors capable of detecting index variations of picoliter (pL) volume specimens therefore offer a promising platform [72]. A micro MZI RI sensor, shown in Figure 13, with a device length of 179.5 μm and consisting of two micro-abrupt-tapers in a cladding-reduced strongly-guiding fibre, was proposed for extremely low volume RI measurements. The cladding of the fibre was etched chemically before being irradiation by a focused CO_2_ laser beam that heated and softened the section of the optical fibre to create a periodic structure. The RI sensitivity was 4000 nm/RIU measured at a wavelength of 1.61 μm over a RI range in the region of 1.45 with a liquid volume of 65.5 pL. Immersion of the sensor in a 72 pL volume of a glucose solution of concentration of 200 mg/mL was shown to cause a red-shift of the channelled spectrum of 0.8 nm and 8 nm with sensitivities of 600 and 4000 nm/RIU at wavelengths of 1.3 and 1.6 μm, respectively [72].

A similar approach, using multi-tapered single mode–multimode–single mode fibre structure, was reported by Zhao, which achieved a sensitivity of 261.9 nm/RIU in the range of 1.3333–1.3737 [73]. In another report, a sensitivity of 500.6 nm/RIU was achieved when the RI varied from 1.333 to 1.411 [74], while Miao reported an RI sensitivity of ∼490.9 nm/RIU over an RI range of 1.3642–1.4015 [75]. 

An interferometric refractometer with variable sensitivity was constructed by placing a single-mode non-adiabatic tapered optical fibre sensor into a fibre loop mirror [76]. The adjustment of the polarization state of the light input to the tapered region, via polarization state controllers (PSCs) inserted in the loop, allowed the excitation of different cladding modes in the taper, resulting in different optical paths for the clockwise and the counter clockwise beams. By variation of the PSCs’ settings, the RI sensitivity of the sensor over the range 1.3380–1.3510 RIU could be tuned from 876 to 1233 nm/RIU.

A modal interferometer consisting of a small core fibre sandwiched between two standard single-mode fibres, with tapers periodically fabricated along the small core fibre using a focused CO_2_ laser beam, has been demonstrated [77]. Measurement of the wavelength shifts of the channelled spectrum features led to a sensitivity of 226.6 nm/RIU in the range from 1.33 to 1.38 RIU.

**Figure 14:**
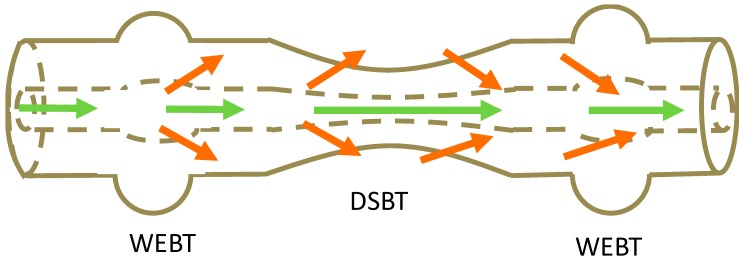
Schematic illustration of the WEBT-DSBT MZI; WEBT, waist-enlarged taper-pair; and DSBT, embedded down-stretching-bitaper (adapted from [21]).

An MZI sensor composed of a waist-enlarged taper-pair (WEBT) (Figure 14) and an embedded down-stretching-bitaper (DSBT), where the waist enlarged tapers excited high-order cladding modes and the DSBT enhanced the evanescent field, was proposed by [21]. By employing the interaction between the strong evanescent field of the high-order cladding mode and the ambient environment, an RI sensitivity of 86.565 nm/cm/RIU was achieved over the RI range from 1.3332 to 1.4140. This sensitivity is about an order of magnitude higher than that of waist-reduced taper-based in-fibre MZIs [21].

In another example of the use of a waist-enlarged taper, a multimode interferometer was created by splicing multimode fibre to single mode fibre (CorningSMF28). The sensor showed a linear response to RI with a sensitivity of −178.424 dB/RIU in the range of 1.351–1.4027 RIU [78].

Abrupt tapers and connector-offset attenuators have been proposed as mode-coupling devices to transfer optical power between core and cladding modes in an optical fibre and to create modal interferometers. The devices were assessed as RI sensors by monitoring the wavelength shifts of features in their channelled spectra [79]. Given a wavelength resolution of 10 pm, an RI resolution of 10^−4^ RIU was reported, similar to that of a cascaded LPG-based MZI.

**Figure 15:**
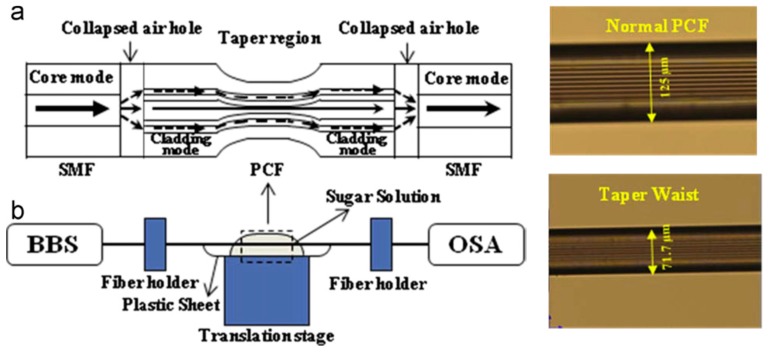
Schematic illustration of the temperature-independent refractometer based on an in-fibre MZI, fabricated by sandwiching a tapered PCF between two standard single mode fibres (CorningSMF28) [80], Reprinted with permission. Copyright 2013 Elsevier.

A temperature-independent refractometer based on an MZI fabricated by sandwiching a tapered photonic crystal fibre (PCF) of length 29 mm between two standard telecommunications SMFs, with the air holes of the PCF fully collapsed in the fusion spliced regions (Figure 15), was investigated [80]. It was found that tapering the PCF greatly enhanced the sensitivity of the refractometer. A maximum sensitivity of 1529 nm/RIU was achieved over an RI range from 1.3355 to 1.4130. The refractometer was found to be nearly temperature-insensitive due to the ultra-low temperature sensitivity of the PCF. In a similar configuration, a sensitivity of 1600 nm/RIU was reported, which is nearly eight times as high as that of an un-tapered PCF interferometer [81] and 20 times higher than that reported for an S-tapered PCF interferometer [82]. A recently reported MZI based on PCF for RI sensing achieved a sensitivity of 1426.70 nm/RIU in the range of 1.3917–1.4204 [83] and 281.6 nm/RIU in the range of 1.3333–1.3737 [84]. 

**Figure 16:**
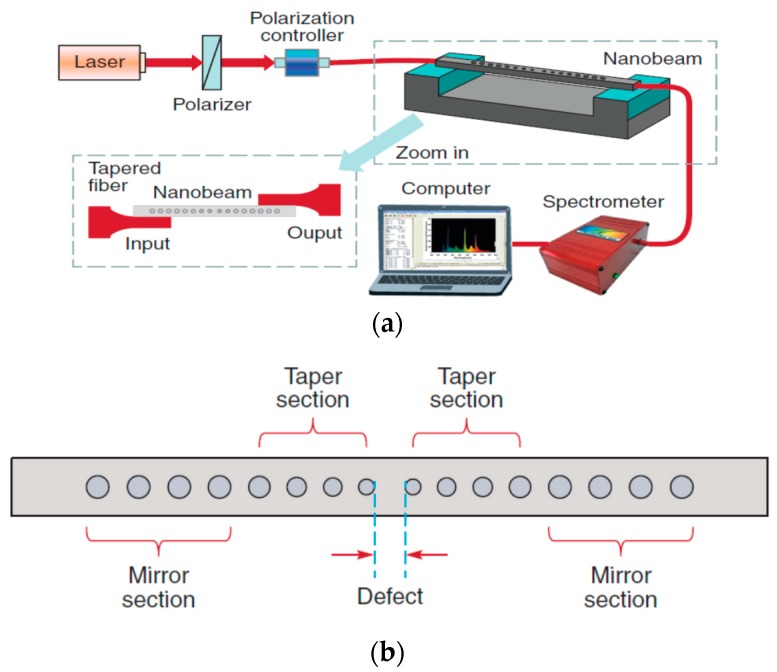
(**a**) Schematic illustration of an optical gas sensor based on a PhC nanobeam cavity; and (**b**) of the PhC nanobeam cavity [85], © Astro Ltd. Reproduced by permission of IOP Publishing. All rights reserved.

A gas sensor based on a photonic crystal nanobeam cavity coupled to a tapered optical fibre was proposed [85]. The nanobeam cavity has seven pairs of tapered air holes and ten pairs of mirror holes (Figure 16) and has a Q factor of 2.2 × 10^6^. A change of the RI of the gas leads to a linear change of resonance wavelength, with a sensitivity of ca. 0.19 nm /10^−^^3^ RIU which could be related to the gas concentration change. 

**Figure 17:**
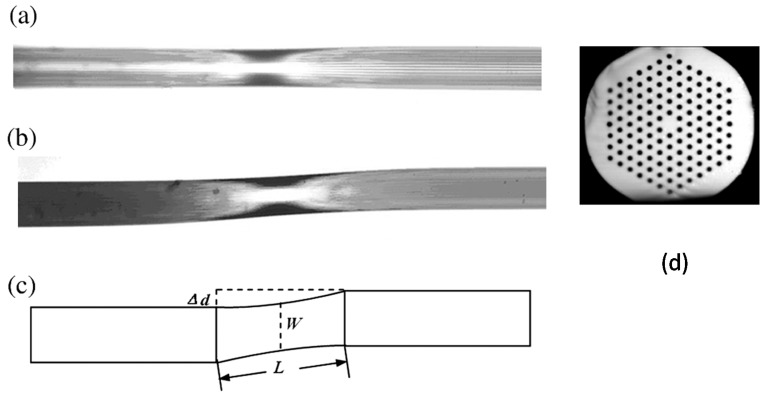
(**a**,**b**) Image of S-tapered PCF; (**c**) sketch of the S-tapered PCF; and (**d**) cross sections of the S-tapered PCF [82].

An S-tapered PCF interferometer (Figure 17), fabricated using a fusion splicer (arc discharge), was shown to have an RI sensitivity of 80 nm/RIU in the range 1.34–1.44, and resolution of 8.5 × 10^−5^ RIU, assuming that a 10 pm wavelength shift can be resolved, with a temperature sensitivity of 4.2 pm/°C [82]. The principle of operation is based on the interference of the fundamental mode with the higher order modes excited by the tapered region of the PCF, where the air holes are collapsed. Since the circularly symmetric character is changed by the imposition of the S-shape, more high-order modes may be excited within the S-tapered section as compared to the straight tapered PCF. On the other hand, the collapsed section is so short that the fundamental mode retains a great part of the total optical power. The S-taper also increases *Δn*_eff_, the difference between the effective refractive indices of the interference modes, which allows the excitation of modes of higher order than can be excited by a straight taper.

In a similar configuration [86] reported a device with a RI sensitivity of 268.8 nm/RIU in the range of 1.332–1.387.

The combination of tapered optical fibres with fibre loop ring down technology was reported by Wu [87]. The sensing principle is based on the dependence on the external RI of the ring-down time, referred to as the time when the light intensity *I* decreases to 1/e of its initial value. Results showed that the sensitivity of this simple scheme could reach −388.581 μs/RIU with the detection limit below 2.57 × 10^−5^ RIU [87]. In another configuration, a Sagnac loop, in combination with a reflective tapered fibre coupler, was used to measure RI [88]. The sensor showed a sensitivity of 3617 nm/RIU for measuring RI in the range 1.33–1.41.

The practical application of a tapered RI sensor was explored recently by Ghahrizjani [89], who demonstrated the measurement of engine oil quality and the time of oil expiration. The oil acts as the external medium for this sensor, and any changes in quality, particle size, and pollution of the oil will influence the optical properties such as the optical power output. By comparing the optical powers between fresh and used oil, the quality of the oil was predicted. Bending of an optical fibre containing two identical tapers at different angles increased the sensitivity to the RI change [90]. It was shown that sensitivities of 106.4 and 126.15 nm/RIU were observed at 45° and 90° bending angles, respectively, for RIs in the range 1.333–1.359.

Table 1 summarizes the performance parameters of the tapered optical fibre based refractometers discussed here. Due to a lack of consistency in the characteristics that are reported in the literature, it is difficult to compare directly different sensors configurations in terms of their performance parameters such as sensitivity, resolution and response time. For example, only one paper [60] reports on the response time. In general, modal interferometers have a higher sensitivity than grating based taper devices and single taper devices. The highest reported sensitivity was 7041.21 nm/RIU [91], but this value was achieved for RIs close to the RI of the cladding. The best RI reported resolution in the biologically relevant RI range was 8.2 × 10^−6^ measured [61]. 

### 3.4. Bio-Chemical Sensors

#### 3.4.1. Evanescent Wave Spectroscopy

Spectral analysis of chemical compounds is an important tool in analytical chemistry that allows selective detection of target compounds in the complex samples consisting of a mixture of the compounds [96]. The simplest implementation of EW spectroscopy using tapered optical fibre could be achieved using a tapering of the multimode optical fibre, which can enhance the performance of evanescent wave spectroscopy-based sensing approaches [97], as it facilitates an increase in the interaction of the evanescent wave with the absorbing compound. 

**Figure 18:**
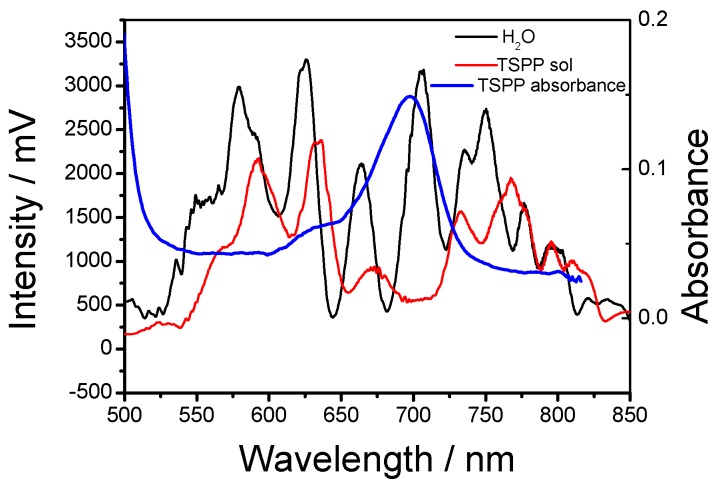
Transmission spectra of a tapered single mode optical fibre (cut-off wavelength 670 nm) with a taper waist of diameter 10 μm and of length 25 mm, measured in: H_2_O, black line, an aqueous solution of Tetrasodium Pyrophosphate Porhine (TSPP) of concentration 10 μM, red line. The absorption spectra of TSPP measured using a conventional UV-vis spectrometer is shown by the blue line [42].

The use of single mode tapered optical fibres allows the simultaneous probing of the spectroscopy and RI of the medium surrounding the fibre, as shown in Figure 18 [42]. As discussed in Section 2.1, in contrast to the transmission spectrum of a cladding removed multimode optical fibre (Figure 4), the transmission spectrum of a non-adiabatic tapered single mode optical fibre consists of a channelled spectrum, arising from the interference of modes excited in the taper, as well as the absorption spectrum of the surrounding medium. Figure 18 shows changes in the central wavelengths of the channelled spectral features of the taper’s transmission spectrum (taper diameter 10 μm, length 25 mm) in response to the change of the RI of a solution of Tetrasodium Pyrophosphate Porhine (TSPP), arising from an increase in concentration, as well as the decrease of the intensity at 700 nm corresponds to the concentration induced changes in the absorption of the TSPP compound. The absorption spectrum of TSPP solution measured using a conventional dual pass spectrometer is also shown in Figure 18.

#### 3.4.2. Fluorescent Sensors 

The first example of the use of a tapered optical fibre sensor for fluorescence spectroscopy was reported in 1996 [98]. The output from an argon ion laser was coupled into the proximal end of a single mode optical fibre with the cut-off wavelength of 450 nm (SM450), which was tapered at the distal end to a diameter of 1 μm. The fabricated probe was modified with the fluorophore compounds (affinity-purified goat anti-human IgG-(H+L) fluorescein isothiocyanate conjugate or affinity-purified goat anti-human IgA (alphachain specific)-tetramethyl rhodamine isothiocyanate (TRITC) conjugate). The fluorescence was excited by the 488 or 514 nm lines of the output from an argon-ion laser that passed through a mechanical chopper. The fluorescence from the bound fluorophores was coupled into the guided mode of the taper. The device offered high sensitivity, 75 pg/mL, and a simple immobilization protocol.

#### 3.4.3. Particle Detection

The detection of airborne particles and aerosols is important in environmental monitoring as it is known that they can be hazardous for the environment and health [99]. 

**Figure 19:**
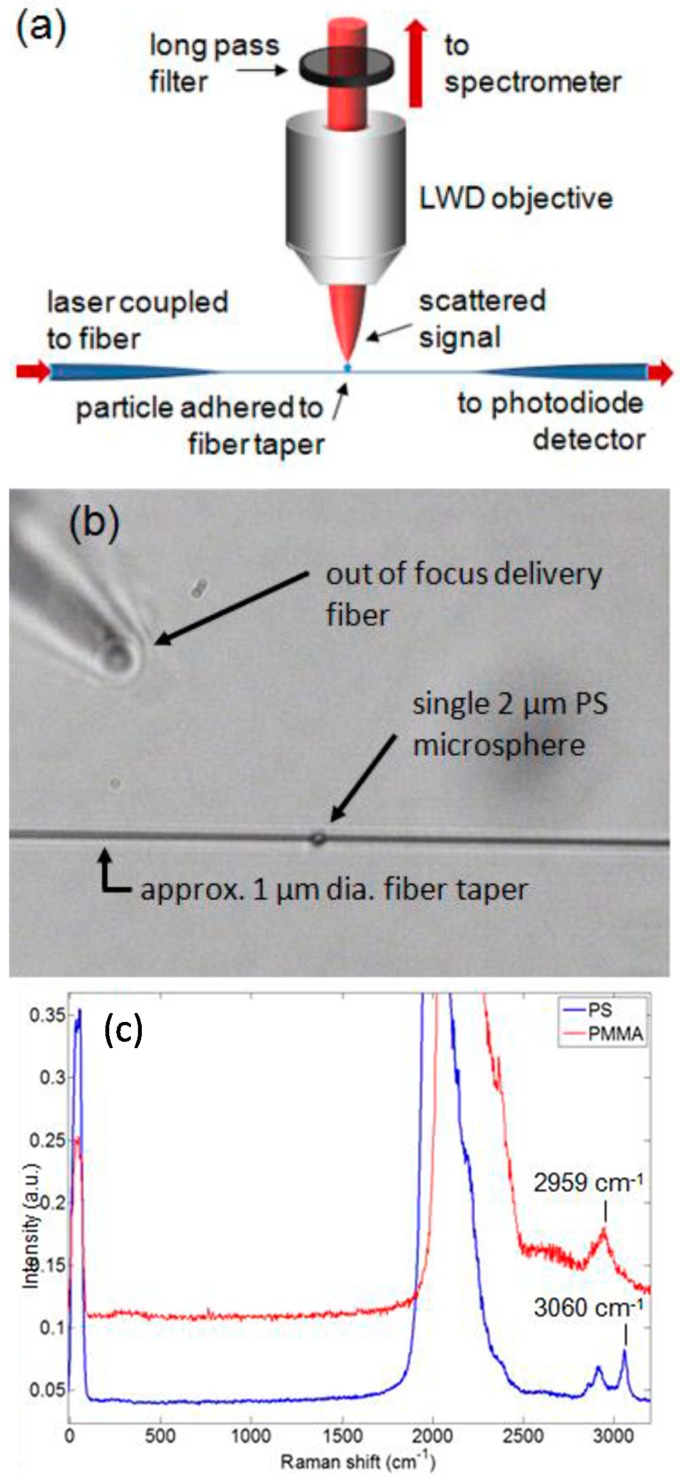
(**a**) Schematic illustration of the experimental set-up; (**b**) image of a 2 μm polymer microsphere delivered and adhered to a 1 μm diameter taper; and (**c**) Raman spectra of 1 μm diameter poly styrene (PS) and Poly-methyl methacrylate (PMMA) microspheres attached to a fibre taper using ca. 760 nm pumping wavelength [31], © 2012 IEEE. Reprinted, with permission).

Tapered optical fibres can be used to facilitate Raman spectroscopy of micro-particles adhered to the surface of the fibre for label-free sensing [31]. The approach is illustrated in Figure 19a, where a particle is in contact with the waist of a fibre taper. The light scattered by the particle from the evanescent wave of the light propagating through the taper is collected in a direction transverse to the optical fibre and the Raman spectrum is analysed. Figure 19b shows an image of a typical experimental configuration, where a 2 μm diameter polymer microsphere is adhered to a fibre taper of 1 μm diameter. The particle was delivered to the taper by another fibre mounted to a three-dimensional stage and was held in place on the surface of the taper by van der Waals forces. Figure 19c shows a comparison of the Raman spectra measured from 1 μm poly styrene (PS) and Poly-methyl methacrylate (PMMA) microspheres individually adhered to the fibre taper (pump laser power ~4–6 mW at 1064 nm, 30 s integration times) [31,100].

**Figure 20:**
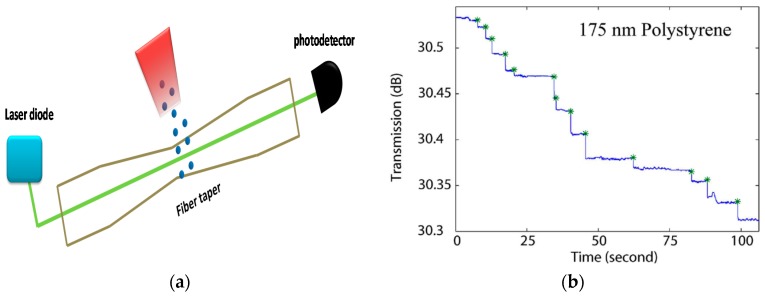
(**a**) Experimental configuration for nanoparticle detection using Rayleigh scattering, (**b**) the changes in the transmission of the tapered fibre as polystyrene nanoparticles bound to a taper of diameter 800 nm and length 33 mm (adapted from [101]. © 2012 IEEE. Reprinted, with permission from [101]).

Rayleigh scattering can be used for nanoparticle detection with single particle resolution [101] as shown in Figure 20a. The sensor consists of a taper of diameter 8 μm and length 3 mm. The sensing method is based on monitoring the transmitted light power, which shows abrupt jumps as single particles bind to the taper surface. The operation of the sensor was demonstrated with polystyrene nanoparticles of radii 120 and 175 nm, as show in Figure 20b. 

It was also shown that single atoms can be detected using a taper of sub-wavelength diameter by detecting single photons spontaneously emitted from the atoms when they were coupled into a single-mode optical fibre with a taper of diameter 400 nm [102]. 

### 3.5. Coated Tapered Devices

It should be noted that the refractometers reported in Section 3.3 were not selectively sensitive. To create a chemically or bio-selective sensor, a thin film that can change its optical properties in response to the presence of the particular analyte has to be deposited onto the surface of the optical fibre. 

#### 3.5.1. Surface Plasmon Resonance (SPR)

Tapered optical fibres facilitate a strong interaction between the propagating modes and the surface plasmon wave generated when the taper is coated with a layer of gold. A number of tapered fibre SPR sensors have been demonstrated. Dı´az-Herrera et al. [103] studied SPR sensors based on doubly deposited uniform-waist tapered fibres (DLUWTs) in which the coating deposited onto the tapered fibres consisted of a metal layer and a dielectric layer. The sensitivity of the DLUWT was shown to be enhanced by increasing the roughness of the optical fibres prior to deposition of the double coating, providing a larger surface area to which to attach the ligands [104].

Verma et al. [95], simulated the behaviour of a tapered fibre sensor in which two identical tapered fibre regions are formed around the metal-coated uniform section of the multimode core sensing region. The study showed that a probe geometry where the ratio of the radii of the fibre core at the input and output ends of the taper lies between 1.5 and 2.0 provided the best performance, with a predicted sensitivity of 15 × 10^3^ nm/RIU for a ratio of 2.0.

Lin et al. [93] employed a tapered fibre coated with noble metal nanoparticles for RI sensing and label-free biochemical detection. The principle of operation of the sensor was based on the evanescent wave excitation of the localized surface plasmon resonance (LSPR), the collective oscillation of conduction electrons confined to metal nanoparticles, whose resonance frequency has been shown to be dependent strongly on the particle’s size, shape, composition, and on the dielectric properties of surrounding medium. The sensing strategy relies on the measurement of the transmission intensity change due to the evanescent field absorption of the gold nanoparticles immobilized on the tapered fibre surface. The RI resolution was 3.2 × 10^−5^ RIU. The feasibility of using an N-(2,4-dinitrophenyl)-6-aminohexanoic acid DNP-functionalized tapered fibre LSPR sensor to monitor anti-DNP antibody was undertaken by spiking a buffer solution with concentrations of antibody ranging from 5 × 10^−9^ to 1 × 10^−6^ g/mL. Results suggested that the compact sensor can perform qualitative and quantitative biochemical detection in real-time [93].

**Figure 21:**
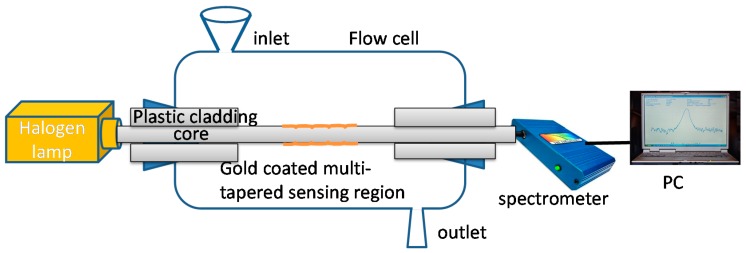
Schematic illustration of the experimental set-up of the multi-tapered fibre optic SPR sensor (adapted from [94]).

Srivastava et al. [94] proposed a more complex approach with a multi-tapered fibre optic SPR sensor, illustrated in Figure 21. The sensitivity was found to increase with increasing number of tapers (from one to eight) and with decrease in taper period from 2.15 to 1.15 mm. An RI sensitivity of 2 × 10^3^ nm/RIU was achieved. 

#### 3.5.2. Functional Coatings

Coating optical waveguides with nanomaterials that exhibit changes in their optical properties upon exposure to targeted chemical species offers the prospect for the development of chemical sensors with high specificity [105,106]. For example, materials such as bromocresol purple [107], oxazine 170 perchlorate [108,109,110] have been used for the development of optical fibre based ammonia gas sensors. 

Generally, the functional materials should have the following properties: transparent in the appropriate spectral range, exhibit changes to their optical properties under the influence of the specific chemical species, have a fast response and have wide dynamic range; be reversible, selective, easy to immobilize onto glass/quartz/plastic fibre and be easily and cheaply manufactured.

A wide range of coating deposition techniques, such as dip- and spin-coating, layer-by-layer deposition (LbL), electrostatic self-assembly, Langmuir–Blodgett deposition and chemical and physical vapour deposition, have been employed for the functional coating of optical fibres [9]. 

The LbL method was used to deposit a multi-layered porphyrin film onto the tapered region of a single mode (Corning SMF28) optical fibre with the aim of demonstrating an ammonia sensor [9]. The coating was composed of alternate layers of tetrakis-(4-sulfophenyl) porphine (TSPP) and poly(allylamine hydrochloride) (PAH). Exposure of the PAH/TSPP coated non-adiabatic tapered optical fibre with a waist diameter of 10 μm to ammonia induced significant changes in the transmission spectrum of the optical fibre. The changes in the coating RI modified differentially the effective indices of the two dominant modes excited in the taper, resulting in a change in phase of the channelled transmission spectrum. The sensor showed a linear sensitivity to the concentration of ammonia in the range of 10–100 ppm, with response and recovery times of order 100 s. The 3σ limit of detection (LoD) was estimated to be ca. 2 ppm. Tiwari et al. [111] demonstrated ammonia sensing with an LoD of 0.1 ppm with a response time of less than 30 s using a nanoscale coating of titanium dioxide, containing a porphyrin as a functional material deposited onto a tapered optical fibre.

In a similar design rabbit anti-goat IgG was immobilised onto an optical fibre taper to provide selectivity towards (tetramethyl rhodamineJisothiocyanate)-labelled goat IgG. The design of the taper proposed by the authors provided a uniform fluorescent signal return along the fibre’s length [112]. Another example of a fluorescence fibre taper sensor used free-base porphyrin self-assembled monolayers (SAMs) that were covalently bonded to the surfaces of a fibre taper and reference glass plates [113]. From fluorescence measurements, the optimum diameter of the coned part of the taper was found to be 35–48 μm for multimode fibres. The mono- and di-protonated forms of free-base porphyrin have distinct fluorescence spectra. This phenomenon was employed to demonstrate a pH sensor operating in the pH range 0.6–3.8.

Tapered optical fibres coated with a ferrocenylenesilylene polymer, [(’5-C_5_H_4_)Fe(’5-C_5_H_4_)MePhSi]_m_, have been used to sense NH_3_ and CO_2_ [114]. The principle of operation of the device is based upon changes in the RI of the polymer layer deposited onto the non-adiabatic taper region of a single mode fibre (Corning SMF28) (tapered region radius is 5.0 μm and a taper elongation of 2.5 mm and an assumed coating thickness of 3.75 μm), which cause a change in power transmitted. An increase in the transmitted intensity was observed when sensor was exposed to CO_2_ gas, while the opposite effect was observed for exposure to NH_3_. The sensitivity of the device increased with the increment of the taper beat length, which is determined by the difference in propagation constants of fundamental and higher order modes, with the response times measured to be in the range of seconds.

A humidity sensor was demonstrated by coating a tapered single-mode fibre (taper waist of 11 μm and length of 1 mm) with a humidity sensitive nanofilm composed of Poly(Diallylmethilammonium chloride) (PDDA) and the polymeric Dye R-478 (Poly-R), deposited using the electrostatic self-assembly (ESA) technique [115,116,117,118,119]. The sensitivity of the device was shown to depend on the optical thickness of the coating, allowing the sensitivity to be optimised by ensuring that it corresponded to the highest slope of the transmitted optical power [115]. The sensing mechanism was based on the measurement of the intensity change induced by the RI change of the humidity sensitive coating. The authors showed that tapers coated with films of higher RI exhibited higher humidity sensitivity [115], as did tapers with thinner waists. The response time of the device was shorter than 300 ms, making this device a candidate for use for the monitoring of human breathing [115]. 

An optical fibre humidity sensor working in reflection mode was demonstrated with a multimode fibre taper spliced between an FBG and the input single mode fibre (Corning SMF28). The taper region was coated with a layer of polyvinyl alcohol that was sensitive to water vapour, while the FBG acted as a reflective filter, reflecting the optical signal back into the taper [120]. Exposure to humidity caused changes in the RI of the coating, with a concomitant change in the transmission of the light signal. The measurement sensitivity was enhanced by passing the light through the taper twice. A maximum sensitivity of 1.994 μW/%RH was demonstrated within the measurement range of 30–95% RH, with a taper waist diameter of 50.2 μm. The average response time was ∼2 s and the measurement was nearly insensitive to temperature with negligible 8 nW change of optical power when temperature changed from 20 to 100 °C.

The coating of a multimode fibre taper interferometer with a polyvinyl alcohol thin film allowed the measurement of relative humidity with a sensitivity of 0.223 nm/%RH in the range of 35–85% RH [121]. By coating an S-shaped tapered optical fibre with SiO_2_ NPs, the measurement of RH with sensitivities of 1.1718 nm/%RH and 0.441 dB/%RH was demonstrated for a humidity range of 83.8–95.2% RH [122]. An optical fibre based MZI coated with ZnO nanowires was used to measure RH in the range of 35–60% RH with a sensitivity of 0.020 nm/% RH [123]. 

The detection of volatile organic compounds using an [Au(PPh_2_C(CSSAuC_6_F_5_)PPh_2_Me)_2_][ClO_4_] vapochromic material incorporated into a sol-gel matrix deposited onto a tapered optical fibre with a waist diameter of 19 μm and of length 1 mm was reported [124]. Changes of up to 13.5 dB in the transmitted optical power were detected on exposure to different concentrations of acetone and dichloromethane vapours. The sensing mechanism relied on the measurement of the decrease of the transmitted power through the tapered fibre caused by a vapour-induced RI change of the coating.

A hydrogen sensor based on the change in absorption of the evanescent field in a 12 nm thick Pd-coated single mode tapered fibre was reported [125,126]. The sensor’s sensitivity was adjustable by means of the taper diameter, interaction length, and/or the wavelength of the light coupled into the fibre. The sensor was capable of detecting hydrogen concentrations below 4% and had a response time of less than 100 s.

A multimode tapered optical fibre modified with a 15 nm-thick Pd thin film was used to detect H_2_. The power transmitted thought the Pd-coated taper (50 μm diameter and 10 mm length) increased when the sensor was exposed to a 2% concentration of H_2_ [125]. Pd films are susceptible to cracking on repeated exposure to hydrogen, which degrades the performance of the sensor. It has been shown that this can be overcome by using Pd–Ag alloy thin films [127]. Another hydrogen sensor was demonstrated by depositing a 150-nm-thick Pd coating over an FBG written in a 50-μm-diameter tapered optical fibre. The sensing head was able to detect H_2_ concentrations in the range 0–1% (v/v) H_2_ at room temperature. A maximum sensitivity of 81.8 pm/% (v/v) H_2_ was attained, with temperature compensation that was achieved by employing a second FBG inscribed in the un-tapered section [128]. An MZI hydrogen sensor with a sensitivity of −1.99 nm/5% of H_2_ was created by the deposition of a 4 nm thick Pd coating onto a tapered optical fibre with a waist diameter of 3 μm [128]. 

A pH sensor was demonstrated by depositing a nanoscale coating of a quinolinium dye onto a tapered optical fibre using the Langmuir–Blodgett technique [8]. It was shown that sensor performance depended on the diameter of the tapered region, with the sensitivity to pH found to be ΔpH = 5 × 10^−^^2^ and ΔpH = 4 × 10^−^^2^ for 4.7 and 37.4 μm diameter fibre tapers, respectively in the pH range of 5–9. The response time was less than 0.5 s, the measurement of which was limited by the integration time of the CCD spectrometer used. 

Tapered optical fibres can also be used as optical probes to measure parameters of interest with spatial resolution on micrometre scales, allowing the direct probing of biological specimens at the cellular level [129]. In a typical fabrication procedure, the end of the optical fibre is tapered to produce a tip with submicron dimensions onto which a thin metal film is deposited, yielding a submicron-sized transmissive aperture at the apex of the probe tip [129]. Such optical fibre tips are used in photon scanning tunnelling microscopes or, when modified with functional materials, to measure chemical and biochemical reactions in microscopic environments [130]. pH sensors of micron and submicron dimensions were developed by immobilising a fluorescent pH sensitive reagent, fluorescein, within a glass film which was deposited on the surface of the fibre tip using a sol-gel process. The probe was used to measure the pH of the intracellular environment of mouse embryonic fibroblast cells [130]. A similar approach was used to develop a pH optrode, using a pH sensitive reagent, 2’,7’-Bis(2-carbonylethyl)-5(6)-carboxyfluorescein, immobilized onto the end-face of taper in a thin xerogel layer [131]. The sensor was employed for local detection of pH in samples simulating native conditions of plant cells, with the measurement based on pH induced changes of fluorescence-intensity spectra. Indium-tin oxide or aluminium coated fibre probes were successfully tested over a pH range from 5.0 to 7.0.

**Figure 22:**
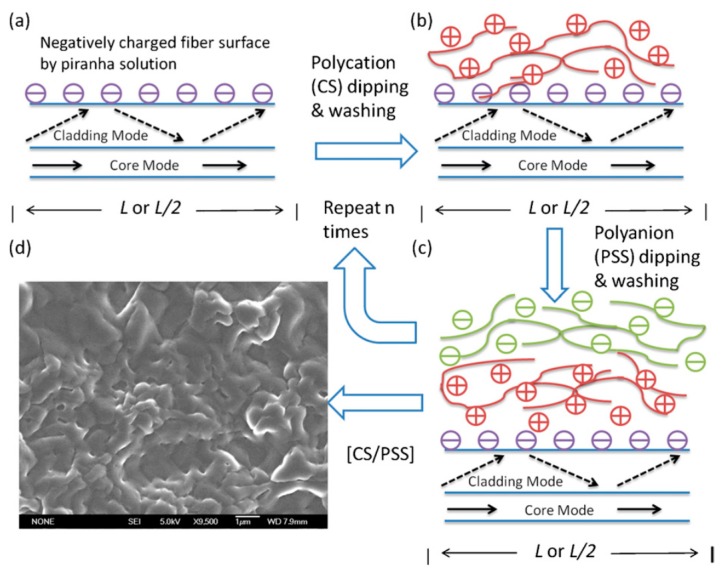
Schematic illustration of the layer-by-layer (LBL) deposition process. Each bilayer deposition constitutes a full cycle of (**a**–**c**). (**d**) The SEM image of multilayer film [CS/PSS] at 1080× magnification [132]. Reprinted with permission. Copyright 2013 Elsevier).

The functionalization of optical fibre modal interferometers based on MZI and MI configurations have been investigated [132]. To endow the sensor with chemical functionality, the surfaces of the fibres were modified with self-assembled polyelectrolyte layers (chitosan (CS)/polysodium styrene sulfonate (PSS)), as shown in Figure 22. Immunoglobulin G (IgG) was immobilized on the polyelectrolyte layer and anti-immunoglobulin G (anti-IgG) molecular binding events were monitored through measurement of wavelength shifts of the channelled spectra of the interferometers. The proposed immunosensors exhibited anti-IgG detection sensitivities of 27.37 nm/(ng/mm^2^) and 5.91 nm/(ng/mm^2^) with concentration detection limits of 0.181 and 4.941 nM for the MZI and MI sensors, respectively. The specificities of the sensors were investigated using correlated/non-correlated anti-IgG–IgG pairs.

In another example, a tapered optical fibre with waist diameter 10 μm and of length 15 mm was modified with antibodies and embedded into a microchannel chip device using micro-electro-mechanical systems (MEMS) fabrication techniques [133]. The sensor was assessed for label-free detection of biomolecules using Immune globulin G (IgG) antibody-antigen pair. 

The deposition of aptamers onto the surface of an optical fibre allows the creation of highly sensitive and selective bio-sensors [134]. For instance, the detection of dopamine with an LoD of 37 nM was achieved using 57-mer dopamine-binding aptamer as the recognition element and a non-adiabatic tapered optical fibre as the probe [134].

Tapered optical fibres modified with nanoscale coatings can also be used for temperature measurements by using thermo-chromic functional materials such as lophine (2,4,5-triphenylimidazole) [135]. A coated fibre taper exhibited a thermal sensitivity of about 0.05 dB °C^−^^1^. It was shown that a narrower taper waist increased significantly the slope of the response curve, and, consequently, the sensitivity of the system. 

The deposition of graphene onto the surface of a tapered POF allowed the measurement of uric acid concentration with a sensitivity of 0.0021 mV/ppm in a range of concentrations between 0 and 500 ppm [136]. However, it should be noted that the device operated as a refractometer, based on the RI change of the solution at the change of the uric acid and the authors did not conduct any selectivity tests. 

In another example [137] reported the use of a tapered fibre coated with metal layers for the detection of pollutants in sea water. Three tapered silica optical fibres, uncoated and coated with metallic (Al or Cu) and dielectric layers (TiO2), were used to determine the presence of oil and hazardous and noxious substances (HNS) in water. The principle of operation was based on the measurement of the RI change induced by the presence of the HNSs. The three tapers with their different coatings were used to cover the wide range (1.329–1.501) of the RI change associated with the pollutants. Several uniform-waist tapers were manufactured with the following geometrical values: total length: 18.3 mm; transition length: 10.98 mm; waist length: 6.34 mm; diameter waist: 40 μm. These tapered devices were coated then with the different coatings to cover the wide RI range (1.329–1.501). Although sensors lacked selectivity toward specific pollutants, the work demonstrated the ability to detect oil and HNS spills in seawater. 

Table 2 summarizes the sensor parameters for the bio-chemical tapered optical fibres reviewed in this section. 

### 3.6. Physical Sensors

In addition to exploiting the access to the EW for chemical and RI sensing, the dependence of the properties of the propagating modes on the mechanical properties of the optical fibre can be used for the measurement of physical parameters such as strain, stress and pressure. 

#### 3.6.1. Strain, Stress and Pressure

Tapers with different taper angles were formed in multimode fibres that were subsequently embedded into glass fibre reinforced epoxy composites. The light transmission characteristics were measured as a function of applied stress. The stress sensitivity of a tapered optical fibre with a 5° taper angle was found to be 0.04 V/GPa. The light intensity transmitted by optical fibre had a linear response as a function of applied stress for different taper angles and the stress sensitivity was shown to increase exponentially with the increase of the taper angle [139].

FBGs inscribed in the waist of tapered fibres offer specific attractive properties for force-sensing applications. A small-diameter fibre reduces its influence on the mechanical properties of the structure for embedded measurements. For tensile forces, the sensitivity scales inversely with the fibre cross-sectional area and it is possible to increase the force sensitivity by several orders of magnitude as compared to FBG sensors in conventionally sized fibres [140]. For tapers with a diameter of 3.5 μm, a force sensitivity of 1900 nm N^−^^1^ can be achieved, which is about three orders of magnitude higher than that of FBGs in conventional 125 μm diameter optical fibres. Due to the small axial stiffness of such tapers, tapered FBGs would be suitable for measurements within thin substrates or in materials of small Young’s modulus, or for the measurements of small forces. 

A fibre optic sensor composed of identical FBGs written on the either side of a tapered cavity was used for temperature independent strain measurement. The sensor possesses two spectral peaks within its main Bragg reflection band acting as an interferometer due to a phase difference between the light reflected by the two gratings. The normalized power difference between the two peaks changes linearly with applied strain but is independent of temperature variation. The accuracy of this particular sensor in measuring strain was estimated to be ±29 με in a range of 1200 με [141]. Bock et al. [53] demonstrated a pressure sensor based on an LPG fabricated using arc discharge to create periodic tapers in a standard single mode fibre, which had a sensitivity of 5.1 pm/bar, an order of magnitude greater than that of a standard FBG.

**Figure 23:**
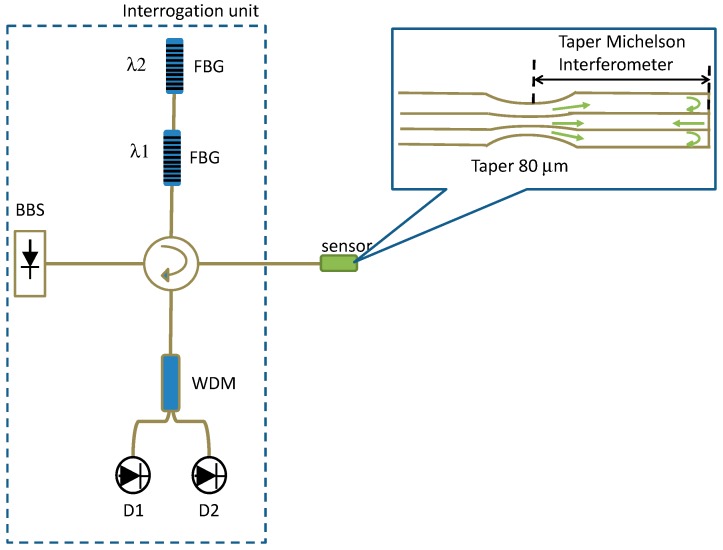
Tapered fibre Michelson interferometer proposed fibre inclinometer. Inset: schematic diagram detail of the fibre-taper Michelson interferometer (adapted [142]).

A fibre taper Michelson modal interferometer consisting of an adiabatic taper of diameter 80 μm located 30 mm from cleaved end of the fibre, as shown in Figure 23, was investigated as a bend sensor in [142]. The bending angle was determined by passive interrogation of the interferometer by generating two quadrature phase-shifted signals from two FBGs with appropriately selected resonant wavelengths (Figure 23). Optical phase-to-bending sensitivity of 0.3 rad/degree and a bend angle resolution of 0.014 degree/√Hz were achieved.

**Figure 24:**
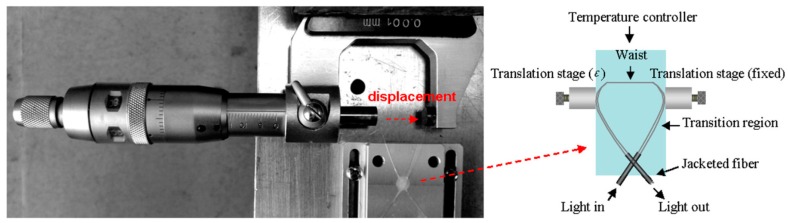
Experimental set-up and configuration of the micro-displacement sensor [143]; IEEE copyright material, Reprinted under creative common free licence (http://creativecommons.org/licenses/by/4.0/).

In another configuration, a temperature-insensitive micro-displacement sensor was demonstrated with a locally bent microfibre taper interferometer (Figure 24) [143]. A change in the bending radius induces a change in the path length of the higher order mode and hence a phase shift between the fundamental and higher order modes. It is possible to control the parameters of the MI, such as its sensitivity and mode coupling conditions, by adjusting the bend- radius. A microfibre taper with a 1.92 μm-waist diameter was optimized by changing the bending radius to minimize the spectral shift of the sensor arising from temperature changes. Through modelling it was shown that the temperature dependence of a locally bent microfibre taper relates strongly to the microfibre taper waist diameter and that it is possible to minimize the temperature dependence over a diameter range from 1.84 to 2.06 μm. The transmission spectrum exhibited a sensitivity of 102 pm/μm.

A similar approach was used to develop an optical fibre inclinometer [144] based on an FBG inscribed in a tapered region of an optical fibre with a measurement range of 0°–90° tilt and an accuracy of ca. 1°. A sensitivity of −4.49 nm/° was achieved using an abrupt taper cascaded with a waist enlarged taper [145].

A noncontact displacement microfibre sensor using a U-shaped adiabatic tapered fibre was proposed with the sensitivity of 0.2 dB/mm operating over the range from 0.6 mm up to 12 mm at a minimum tapered diameter of 8 μm [28]. The use of a fibre modal interferometer for local lateral compression distance detection was described in [146]. The transmission spectrum of the interferometer was measured under different transverse pressures and with the loading at different locations along the interferometer. The spectra were analysed using a fast Fourier transform with the amplitude and spatial frequency of different peaks in the spatial frequency spectrum corresponding to different cladding modes can be utilized to evaluate local fibre geometry deformation. The sensitivity to lateral compression distance was found to be determined by the fibre taper structure (such as waist diameter and its length) with values of 9.996 × 10^−^^6^ dBm/μm in the range from 7.5 to 27.5 μm. An MZI constructed by connecting a LPG with an up-taper was used to measure strain in the range of 0–590 με, with a sensitivity of 0.026 dB/με, was reported in [147]. 

Interferometric measurement configurations can also be implemented using PCF. A tapered PCF interferometer and a microhole-collapsed PCF interferometer have been used for the detection of interaction forces generated in surgical devices, the key feature is that they use of PCF means that they are inherently immune to the influence of ambient temperature variation [148]. The devices were used for force characterization in laparoscopic scissor and standard surgical scissor blades. It was found that PCF-instrumented surgical blades outperformed blades fitted with the FBG sensors during static load measurement, with the strain sensitivity of 1.68 pm/με for PCF sensor and 1.2 pm/με for the FBGs. 

#### 3.6.2. Temperature

**Figure 25:**
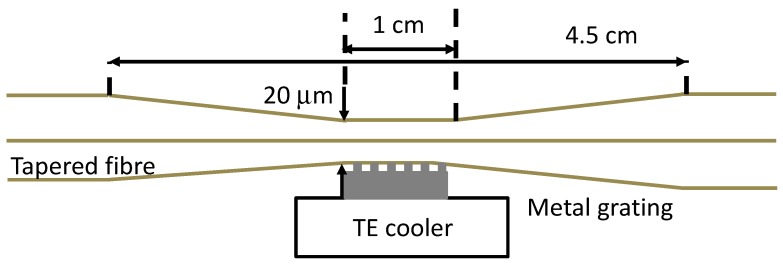
Schematic illustration of an LPG composed of a tapered optical fibre with a uniform waist and a side-contacted metal grating (adapted from [57]).

An LPG induced in a tapered optical fibre attached to a heated metal grating, as shown in Figure 25, was studied [57]. The temperature and RI sensitivities were enhanced compared with LPGs of the same period fabricated in standard optical fibres, and the unusual spectral response of decreasing phase-matching wavelength with respect to increasing grating period and temperature was observed. The temperature and RI sensitivities were measured to be 0.24 nm/°C and 650 nm/RIU, respectively.

An optical fibre MZI consisting of two waist enlarged tapers with a temperature sensitivity of 0.070 nm/°C for a 7.5 mm long interferometer was reported by [149]. A strain sensor was demonstrated using a MZI composed of two abrupt single-mode fibre tapers exhibiting a sensitivity of 2000 nm/ε similar to that of LPG sensors, but simpler to manufacture [150]. An MI can be built with tapered large mode-area microstructured optical fibre (MOF). Such a fibre had the following parameters: core diameter of 11 m, average hole diameter of 2.7 μm, average hole spacing (pitch) of 5.45 μm, and outside diameter of 125 μm. The tapering was used to collapse the air holes such that, in the taper waist, the fibre was transformed into a solid unclad multimode fibre. This allows the coupling between the fundamental HE_11_ MOF mode and the HE_1_ modes of the solid fibre. The interferometer was used to demonstrate a wavelength-encoded temperature sensor, showing a linear response over a temperature range from 200 to 1000 °C [151]. An S-tapered fibre MZI created in an all-solid photonic bandgap fibre was used to measure temperature in the range of 20–80°C with a sensitivity of 49.52 pm/°C and low sensitivity to strain of 0.455 pm/με [152]. Measurement of temperatures up to 800 °C was demonstrated by splicing a thin core optical fibre to a length of SMF and fabricating an up-taper at the splicing point, forming an MI sensor. A sensitivity of 0.140 nm/°C was achieved in the range of 30–800 °C. 

#### 3.6.3. Simultaneous Measurements of Two or More Parameters

As mentioned previously, the inscription of a grating within a taper section of optical fibre, or the tapering of a region already containing a fibre grating, can facilitate multi-parameter measurements, where two or more parameters are measured using the same device. Generally, the principle of simultaneous measurement of two or more parameters utilises the difference in sensitivity of the sensor to these parameters. A simple matrix operation allows determination of the desired parameters.

Simultaneous measurement of strain and temperature was demonstrated using a periodically micro-tapered fibre grating (period of 1.3 mm and the diameter of the tapered region was 76 mm), where the resonance wavelength was blue-shifted and the transmission decreased with increasing strain, while the opposite was observed for increasing temperature. Consequently, it was possible to discriminate the influences of strain and temperature with the sensitives of –0.55 nm/με and 49.6 pm/°C by measuring the resonant wavelength and the transmission with the sensitives of −0.32 dB/ με and −0.01 dB/°C [153]. 

**Figure 26:**
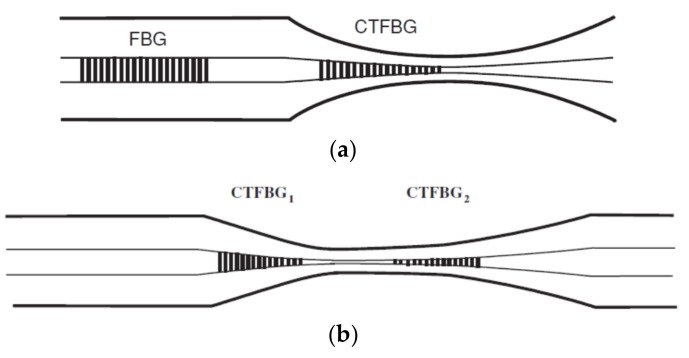
(**a**) Sensing head geometry with FBG/chirped tapered FBG (CTFBG) structure; and (**b**) sensing head geometry using two CTFBGs in biconical taper structure [155]. © IOP Publishing. Reproduced by permission of IOP Publishing. All rights reserved).

FBGs have been combined with tapers to facilitate the measurement of temperature and strain by writing the FBG in a linearly etched fibre that provided information encoded in the peak wavelength and in the spectral width of the FBG. The spectral width of the grating depended uniquely on the applied strain and was temperature independent. An uncertainty of ±15.26 and με ±1.92 °C was achieved [154]. In a more complex configuration, the FBGs were written in different sections of the taper, as illustrated in Figure 26. In the first configuration, FBGs of uniform period were fabricated in the untapered and tapering sections, as shown in Figure 26a. The effective index of the propagating mode changes along the tapering section, introducing a chirp to the grating and broadening the Bragg spectrum, while the change in diameter offers a variation in strain sensitivity along the grating length, allowing an applied strain to influence the chirp. The second sensing head consists of FBGs written the two tapering regions, where these regions have different taper angles, providing a larger difference in the strain sensitivity of the structure. The second structure offers a higher sensitivity compared to the first sensing head with strain and temperature sensitivities determined to be 1.81 ± 0.03 pm/με and 9.67 ± 0.09 pm/°C, respectively [155].

**Figure 27:**
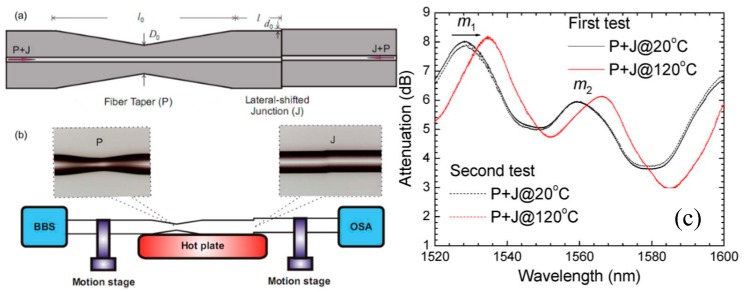
(**a**) Schematic illustration of the a device consisting of tapered fibre with a lateral-shifted junction; (**b**) experimental setup with the left and right insets showing the optical micrographs of the fibre taper (P) and the lateral shifted junction (J) in the interferometer, respectively; and (**c**) attenuation spectra of asymmetric interferometer at different temperatures (adapted from [156]).

An asymmetric fibre MZI consisting of a fibre taper and a lateral-shifted junction (Figure 27) was shown to allow the simultaneous measurement of axial strain and temperature in [156]. The interferometer exhibited different environmental sensitivities for different device architectures. If the taper and the lateral-shifted junction (P+J in Figure 27a,b) were located in close proximity (<15 mm), then the device exhibited temperature sensitivities of 60.4 and 63.9 pm/°C (red shift) and axial strain sensitivities of –1.47 and –2.71 pm/με (blue shift) for the higher and lower interference orders *m*_1_ (49) and *m*_2_ (48), respectively (Figure 27c). The junction–taper interferometer (J+P in Figure 27a) has temperature sensitivities of 60.1 and 63.3 pm/°C (red shifts) and axial strain sensitivities of −1.51 and −2.75 pm/με (blue shifts) for the interference orders *m*_1_ and *m*_2_, respectively.

**Figure 28:**
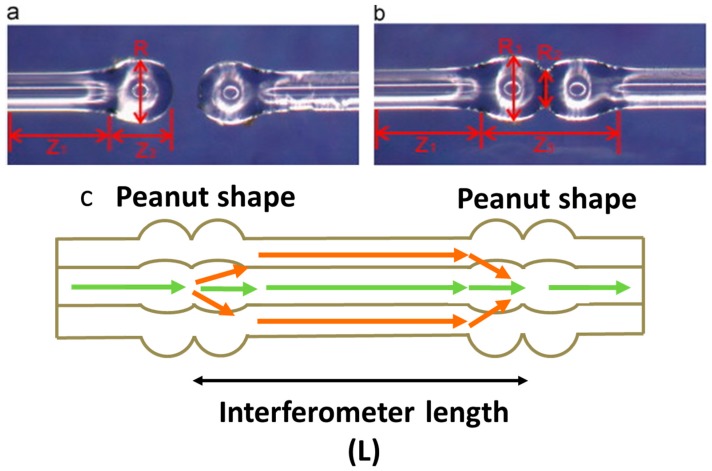
Microscopic image of the peanut-shape structure: (**a**) after the arc discharge treatment and (**b**) after the fusion splicing. Z_1_, distance to ellipsoidal-structure section Z_2_, length of ellipsoidal-structure section; R, radius of the ellipsoidal-structure section; (**c**) schematic diagram of the cascading two waist enlarged structures-MZI (adapted from [157]).

By cascading two waist enlarged structures that facilitate coupling and recoupling between the fibre core mode and the cladding modes, a simple and low-cost MZI was demonstrated (Figure 28a,b) [157]. The first waist enlarged structure coupled the core mode to cladding modes, while the second recouples the light from the cladding modes into the core mode, producing a high visibility channelled spectrum. For an interferometer of length 22 mm, the temperature sensitivity of the device was 46.8 pm/°C and the strain sensitivity was 14 pm/με. Using a similar configuration, sensitivities of 57.5 pm/°C and 1.02 pm/με for temperature and strain, respectively, were achieved [158]. 

In another example of the use of MI, the simultaneous measurement of axial strain and temperature was demonstrated by introducing a 20 × 4 μm spindle-shaped spot produced by a femtosecond laser and located 30 mm away from the fibre taper (with the diameter and length of 60 and 600 μm respectively) in a standard single-mode optical fibre. The spot in the optical fibre can be regarded as a Mie scattering centre where the fundamental core mode will be scattered into higher order cladding modes. This results on the interference between the light coupled by taper and light coupled to higher order modes by a spindle-shaped spot. The experimental results indicate axial strain sensitivities of −1.0 and −1.2 pm/με (blueshift) and temperature sensitivities of 81.3 and 98.8 pm/°C (redshift) at 1513.4 and 581.2 nm, respectively, corresponding to two interference orders [159]. 

The simultaneous measurement of strain and temperature with resolutions of ±5.6 με and ±1.6 °C and sensitivities as high as −23.69 pm/με for a 15-μm diameter taper, respectively, was achieved using tapering single-mode–multimode–single-mode structures [160].

The use of an MZI realized in a tapered single-mode optical fibre was proposed for the measurement of RI and temperature [161]. The features of the channelled spectrum shift with changes in the surrounding RI and temperature. Simultaneous measurement of RI and temperature with corresponding sensitivities of up to 26.087 nm/RIU (blueshift) and 0.077 nm/°C (redshift) was achieved. In other work, the RI and temperature sensitivities for a device comprising two bi-tapers and an LPG were 108.16 nm/RI and 0.12 nm/°C, respectively [92].

It was shown that it is possible to measure simultaneously three parameters, such as RI, force and temperature, by monitoring different interference peaks of the transmission spectrum of an MZI based on a tapered hollow core optical fibre [91]. The sensor demonstrated an RI sensitivity of about 7041.21 nm/RIU in the range of 1.4406∼1.4458, a stress sensitivity of −6.26 nm/N in the range of 0∼1 N and a temperature sensitivity of 9.8 pm/°C in the range of 30∼100 °C. 

**Figure 29:**
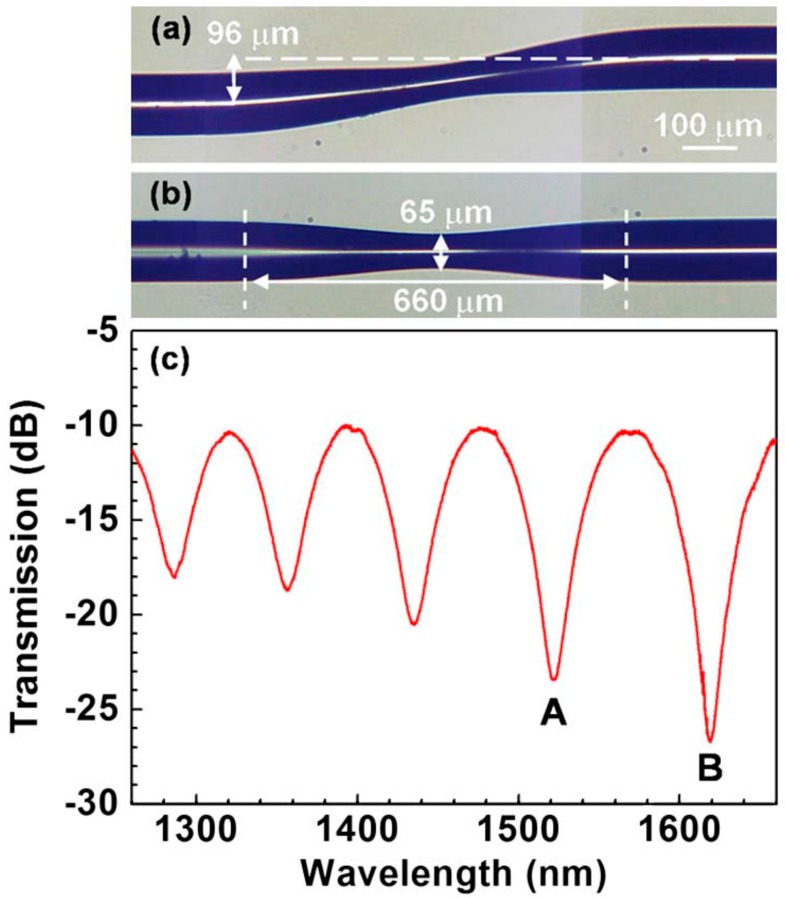
(**a**) Side view of the S-tapered fibre (SFT) in optical microscopy. (**b**) Top view of the SFT. (**c**) Transmission spectrum of the single SFT MZI in air. (Reprinted with permission from [162]).

An MZI with a length of hundreds of microns was demonstrated based on an S-tapered fibre (SFT), shown in Figure 29 [162]. The dependence of the spectral characteristics of the SFTs on the axial offsets and diameters has been studied. An SFT with an axial offset of 114 μm and a taper waist diameter of 54.6 μm was found to exhibit the best sensitivity to RI, with a sensitivity of 2066 nm/RIU in the 1.407–1.421. The strain sensitivity was –183.4 pm/με when the axial offset of the SFT was 138 μm and the taper waist diameter was 65.0 μm. By embedding S-tapered optical fibres into a polydimethylsiloxane patch, such that the patch transduced a transverse load into an axial strain, measurement of load and temperature with sensitivities of −29.03 nm/N and −2.17 nm/°C were reported. 

Temperature-independent strain and angle measurements are achieved using a taper fabricated on a Bragg fibre using a CO_2_ laser [163]. Bragg fibres are cylindrical waveguides consisting of a low-index core surrounded by concentric rings of material with alternating high and low RI, acting as a cylindrical Bragg mirror. The sensitivity was 22.68 pm/με and 185.10 pm/deg to strain and angle, respectively. Another interesting approach to the measurement of several parameters simultaneously employed a heterogeneous multicore optical fibre, where in one fibre there are several cores, each having different propagation properties [164]. Tapering such a fibre allowed the demonstration of a temperature sensitivity of 47.37 pm/°C for the central core and 53.20 pm/°C for the outer core, with a strain sensitivity of 1.10 pm/με for the central core and 0.84 pm/με for the outer core. Different spatial channels (cores) in heterogeneous multicore fibre have dissimilar responses to the outer environment allowing multiparameter measurements. Using temperature-strain matrix, the measured relative errors were estimated to be less than 5%. Table 3 summarizes parameters of the tapered sensors for physical measurands.

## 4. Conclusions 

A review of the uses of tapered optical fibres as sensors has been presented. The theory of light propagation in the tapered optical fibres and methods for their fabrication was discussed briefly. The measurement of physical and chemical parameters using a variety of optical fibre tapers configurations has been outlined. Despite clear advantages, such as high sensitivities and simplicity of fabrication, the wide variety of configurations and their versatility, there are limited reports of practical application of the tapered optical fibres. The majority of the work that has been conducted is laboratory-based in well-controlled environments. One of the major challenges for future applications of tapered optical fibre sensors lies in the reliable and reproducible fabrication of the devices. It should be also noted that in simplest form tapered devices are not selectively sensitive and require coating that provide specificity. In addition, addressing issues around packaging, handling and mechanical strength is essential.

## Figures and Tables

**Table 1:** Summary of the parameters of the tapered based RI sensors.

Type of the Sensor	Taper Geometry	Sensitivity/RIU Resolution RIU	RI Range	Reference
Single taper devices	Waist 60 μm	10^−4^ RIU	1.36–1.46	[43]
	Waist 7 μm	8.2 × 10^−^^6^	1.3330–1.3447	[61]
Mie scattering (silica nanoparticles)	Waist 2.8 μm	1.8 × 10^−^^5^ RIU	1.32–1.46	[63]
LPG	Waist 34 μm LPG 400 μm	1 × 10^−4^ RIU	1.30–1.34	[65]
	Waist 20 μm LPG ca. 400 μm	650 nm/RIU	1.00–1.36	[57]
	Waist 120 μm LPG 380 μm 6 periods	372 nm/RIU	1.33–1.46	[55]
	Waist 55 μm Length 3 mm	490.9 nm/RIU	1.3642–1.4015	[75]
MZI	Waist 25 μm LPG 300 μm	8.5 *×* 10^−^^5^	1.330–1.335	[64]
	Waist 63.75 μm LPG 414 μm	5.8 × 10^−6^ RIU	1.332–1.362	[67]
	Waist 54.6 μm LPG 564 μm	2066 nm/RIU	1.407–1.421	[30]
	Bi-taper length and waist 165 and 278 μm LPG period 550 μm and length 30 mm	−108.16 nm/RI	1.338–1.363	[92]
	Waist 66.5 μm Length 309 μm	7041.21 nm/RIU	1.4406–1.4458	[91]
FBG	Waist 30 μm	2.5^−5^ × 10^−5^	1.450–1.456	[68]
	-	1.63 ± 0.0 × 10^5^ 3.05 ± 0.01 × 10^5^ dB/RIU	-	[70]
	6–8 μm	876–1233 nm/RIU	1.3380–1.3510	[76]
	Waist 90 μm LPG 500 μm	226.6 nm/RIU	1.33–1.38	[77]
	UFBT 167 μm DSB 82 μm	86.565 nm/cm/RIU	1.3332–1.4140	[21]
	Waist 40 μm	10^−4^ RIU	1.315–1.3618	[46,79]
Photonic crystals	Waist 71.7 μm	1529 nm/RIU	1.3355–1.413	[80]
	Waist 30 μm	1600 nm/RIU	1.3333–1.3577	[81]
	Waist 2 μm	190 nm/RIU	1.000–1.009	[85]
	Waist 60 μm	8.5 × 10^−5^	1.34–1.44	[82]
	Waist 70 μm	1426.70 nm/RIU	1.3917–1.4204	[83]
	Length 440 μm Waist 284 μm	281.6 nm/RIU	1.3333–1.3737	[84]
SPR	Waist 48 μm	3.2 × 10^−5^ RIU	1.33–1.40	[93]
	-	2 × 10^3^ nm/RIU	1.335–1.380	[94]
	Waist 300 μm	15 × 10^3^ nm/RIU	-	[95]
Multi-taper devices	Waist 52 μm Length 552 μm	261.9 nm/RIU	1.3333–1.3737	[73]
	Waist 76.5 μm and 13.2 μm	4000 nm/RIU	around 1.45	[39]
	Waist 17 μm Length 3 mm	500.6 nm/RIU	1.333–1.411	[74]
Bending at 45° and 90°	Waist 40 to 50 μm	126.15 nm/RIU at 90^o^	1.333–1.359	[90]
Fibre Loop Ring Down Technology	Waist 28.2 μm Length 728 μm	−388.581 μs/RIU	1.3333–1.3737	[87]
Sagnac loop	Waist 18, 13 and 9 μm Length: 16, 17 and 18 mm	3617 nm/RIU	1.33–1.41	[88]
Michelson interferometer	Length 20cm	1.35 × 10^4^/RIU	1.3330–13470	[71]
	Waist 165–185 μm Bi-taper length is 375–490 μm Device length 20 mm	−178.424 dB/RIU	1.351–1.4027	[78]

**Table 2:** Summary of the parameters for bio-chemical tapered optical fibre sensors.

Analyte	Taper Geometry/Waist Diameter	Sensitivity Limit of Detection	Coating Material	Response/Recovery Times	Dynamic Range	Reference
Ammonia gas		detection limit of 10 ppm	bromocresol purple	Response time 5 min Recovery time 20 min	10–1000 ppm	[107]
		LoD 15 ppm	Oxazine 170	-	0–3000 ppm	[108]
	Waist 10 μm	LoD 2 ppm	Porphyrin TSPP	Response 100 s recovery 240 s	10–100 ppm	[110]
	Waist 41 μm	0.05%/Torr	ferrocenylenesilylene polymer, [(’5-C5H4)Fe(’5-C5H4)MePhSi]_m_	ca. 5 min	6–350 Torr	[114]
	Waist 17 and 40 μm Length 4.5 mm	LoD 0.1 ppm	Porphyrin TMPyP incorporated into TiO_2_	Response 30 s	0.1–10,000 ppm	[111]
pH	Waist 35 μm	-	porphyrin	-	0.6–3.8	[113]
	5 μm 37.4 μm	ΔpH = 5 × 10^−^^2^ ΔpH = 4 × 10^−^^2^	quinolinium dye	0.5 s	2–10	[8]
	30 μm	0.05 units of pH	poly (allylamine hydrochloride) and poly(acrylic acid)	60 s	4.0–6.0	[119]
	4 μm 60 μm	-	2’,7’-Bis(2-carbonylethyl)-5(6)-carboxyfluorescein	-	5.0–7.0	[131]
CO_2_	Waist 41 μm	0.06%/Torr	ferrocenylenesilylene polymer, [(’5-C5H4)Fe(’5-C5H4)MePhSi]_m_	ca. 5 min	6–350 Torr	[114]
RH			Poly(Diallylmethilammonium chloride) (PDDA) and the polymeric Dye R-478 (Poly-R)			[115,116,117,118]
	Waist 50.2 μm	1.994 μW/% RH	polyvinyl alcohol	ca. 2 s	30–95% RH	[120]
	Waist 150 μm	0.223 nm/% RH	polyvinyl alcohol (5 μm)	-	35% to 85%	[121]
	Waist 74 μm Length 468 μm	1.1718 nm/% RH and 0.441 dB/% RH	SiO2 NPs (7 μm)	-	83.8% RH to 95.2% RH	[122]
	Waist 95 μm Length: 1 mm	0.020 nm/% RH	ZnO	-	35–60% RH	[123]
acetone	19 μm	0.04 dBm/mm Hg	[Au(PPh_2_C(CSSAuC_6_F_5_)PPh_2_Me)_2_][ClO_4_] vapochromic material	32 min	231–277 mm Hg	[124]
dichloromethane	19 μm	0.03 dBm/mm Hg	[Au(PPh_2_C(CSSAuC_6_F_5_)PPh_2_Me)_2_][ClO_4_] vapochromic material	31 min	436 mm Hg	[124]
H_2_	20 μm	0.05 %/% H_2_	Pd-coated	<100 s	0–10.5%	[125,126]
	50 μm	0.02 %/% H_2_	Pd-coated	<100 s	0–2%	[60]
	36 μm	0.01 625 mW/% H_2_	Pd–Ag alloy	-	0–4%	[127]
	50 μm	81.8 pm/% (v/v) H_2_	Pd coating	-	0–1% (v/v) H_2_	[128]
	5 μm	–1.9 nm/5% H_2_	Pd coating	10 s	0–5% (v/v) H_2_	[138]
Biosensor	146 μm	0.181 nM	Immunoglobulin on chitosan (CS)/polysodium styrene sulfonate (PSS))	100 s	2–11 nM	[132]
Uric acid	POF (waist 0.45 mm)	0.0021 mV/ppm	Graphene in PVA	-	0–500 ppm	[136]
dopamine	Length 15–20 mm; waist 6–9 μm	LoD 37 nM	Dopamine-specific DNA aptamer	-	0–1 μM	[134]

**Table 3:** Summary of the parameters of the tapered sensors for physical measurands.

Measurand	Taper Geometry	Sensitivity Limit of Detection	Dynamic Range	Reference
Temperature	Waist 20 μm LPG ca. 400 μm	–0.24 nm/°C	25–75 °C	[57]
	170 μm 280 μm	0.070 nm/°C	0–450 °C	[149]
	-	46.8 pm/°C	-	[157]
	Waist 66.5 μm Length 309 μm	9.8 pm/°C	30∼100 °C	[91]
	Waist 97 μm Length 491 μm	49.52 pm/°C	20–80 °C	[147]
	Waist 42 μm Length 2.4 mm	47.37 pm/°C	20–80 °C	[164]
	Waist 168 μm Length 245 μm	57.5 pm/ °C	25–70 °C	[158]
	Waist 165 μm Length 340 μm,	0.140 nm/°C	30–800 °C	[152]
Stress	Taper angle 5^o^	0.04 V/GPa	0–0.5 GPa	[139]
	Waist 66.5 μm Length 309 μm	−6.26 nm/N	0∼1 N	[91]
	-	1.2 pm/με	1200 με	[141]
Strain	40 μm	2000 nm/ε	100–900 με	[150]
	-	14 pm/με	-	[157]
	65 μm	–183.4 pm/με	-	[162]
	Length 5 mm waist 35 μm	22.68 pm/με	0–400 με	[163]
	Waist 168 μm Length 245 μm	1.02 pm/με	81.3–1626 με	[158]
	Waist 161 μm	0.026 dB/με	0–590 με	[147]
Force	4 μm	1900 nm N^−^^1^	0–0.15 N	[140]
Pressure	115–120 μm	5.1 pm/bar	0–450 bar	[53]
Angle	50 μm Length 44 mm	1°	0°–90°	[144]
	Length 5 mm waist 35 μm	185.10 pm/deg	0°–10°	[163]
	Length 1.37 mm waist 50 μm	−4.49 nm/°	3°–6.66°	[145]
